# Neural Network-Based Filter Design for Compressive Raman Classification of Cells

**DOI:** 10.1021/acs.jcim.3c01856

**Published:** 2024-07-03

**Authors:** Stefan Semrau

**Affiliations:** Leiden Institute of Physics, Leiden University, Leiden 2333CA, The Netherlands

## Abstract

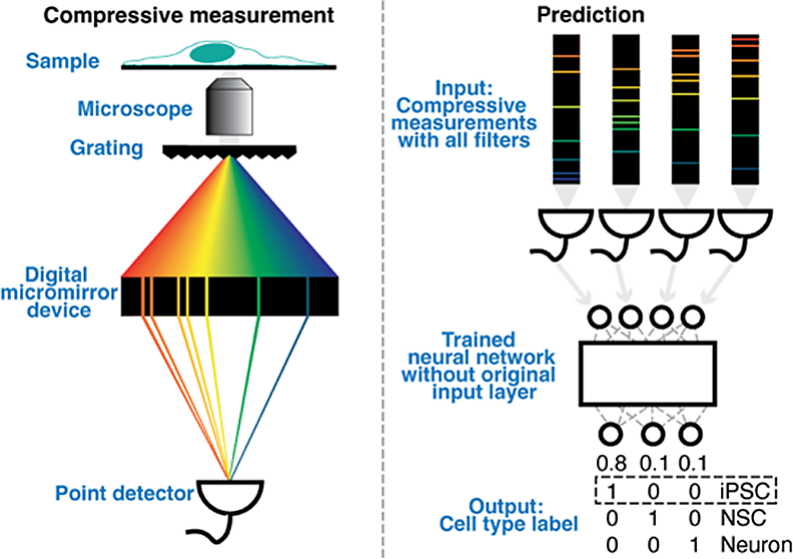

Cell-based therapies are bound to revolutionize medicine, but significant technical hurdles must be overcome before wider adoption. In particular, nondestructive, label-free methods to characterize cells in real time are needed to optimize the production process and improve quality control. Raman spectroscopy, which provides a fingerprint of a cell’s chemical composition, would be an ideal modality but is too slow for high-throughput applications. Compressive Raman techniques, which measure only linear combinations of Raman intensities, can be fast but require careful optimization to deliver high performance. Here, we develop a neural network model to identify optimal parameters for a compressive sensing scheme that reduces measurement time by 2 orders of magnitude. In a data set containing Raman spectra of three different cell types, it achieves up to 90% classification accuracy using only five linear combinations of Raman intensities. Our method thus unlocks the power of Raman spectroscopy for the characterization of cell products.

## Introduction

Spurred by spectacular successes, cell-based therapies continue to be a very active field of research.^1^ As therapeutic agents, cells enable unique applications and, in some cases, greatly surpass traditional drugs in efficacy. For example, mesenchymal stem cells, which can differentiate into multiple cell types and have immunomodulatory effects,^2^ have been explored as therapeutic agents in arthritis^3^ and other diseases.^4^ Chimeric antigen receptor T (CAR T) cells have produced remarkable results in hematological cancers and might revolutionize personalized cancer therapy.^5^ Finally, the ability of induced pluripotent stem cells (iPSCs) to differentiate into various cell types has been exploited in recent clinical trials aiming to repair heart muscle tissue or the cornea.^6^ The unique capabilities and tremendous potential of cell-based therapies come with a challenging production process.^7^ Whereas the manufacture of small molecule drugs can be easily standardized, automated, and scaled up, cell products typically require manual labor, suffer from intrinsic heterogeneity, and are difficult and laborious to optimize.^8,9^

One reason for these difficulties is the lack of quantitative, noninvasive readouts of a cell’s state during and after the production process.^10^ Currently, quality control of cell products occurs in a sample of the final product,^11^ which is problematic for several reasons. If quality control fails, the typically lengthy production process has to be repeated, which might delay a time-critical therapy. Additionally, the cells that are sampled for quality control cannot be used for therapy as existing measurement methods are destructive. Since there is considerable heterogeneity at the single-cell level^12,13^ the—necessarily untested—therapeutic product might contain cells that are harmful to the recipient. For these reasons, there is an urgent need for nondestructive measurement methods that can assess the state of all cells in a population without exogenous labels or manipulations that might have a negative impact on safety. Ideally, the method should also be contactless, allowing for the use of closed-system manufacturing, which can reduce costs and the risk of contamination.^14,15^ Just like the measurement of pH and temperature enables the closed-loop control of fermentation in a bioreactor, access to a cell’s state in real time would allow us to control and optimize the production process of cell-based therapies. This would greatly reduce variability, improve the safety profile, and reduce costs.

The requirement to be nondestructive and label-free narrows down the choice of potential readouts to optical or electrical modalities. Light microscopy has been used extensively to assess cell morphology and can detect the early onset of cell differentiation when coupled with deep learning.^16^ Electrical impedance is used routinely to measure cell viability and is currently explored in assays of cell adhesion and differentiation.^17^ Notwithstanding the usefulness and importance of these techniques, their information content is limited and insufficient to characterize a cell’s state comprehensively. Spectroscopic methods can in principle provide significantly more information. For example, autofluorescence spectroscopy can reveal useful information about a cell’s metabolic state, but it is restricted to molecules that autofluoresce.^18^ Autofluorescence spectra of particular molecular species also tend to be broad, which makes them difficult to unmix. Raman spectra, on the other hand, can be collected from a large set of molecules ranging from carbohydrates over lipids to nucleic acids and proteins.^19^ A Raman spectrum of a cell is therefore essentially a fingerprint of the cell’s chemical composition. Unsurprisingly, it has been used in regenerative medicine applications.^20,21^ Unfortunately, the spontaneous Raman effect, which is due to the inelastic scattering of light, has low efficiency and the intensity of the scattered light is therefore low. Consequently, collecting enough photons to obtain a complete Raman spectrum covering all relevant molecular species takes time and precludes high-throughput applications.

One possible solution to this problem is provided by compressive sensing.^22−24^ In this approach, only a few linear combinations of Raman intensities at certain wavenumbers are measured, thereby greatly reducing measurement time. Mathematically, a compressive measurement is a dot product between two vectors: the complete Raman spectrum and a filter vector. For technical reasons related to the implementation of the measurement with optical elements, the filter vector is subjected to constraints. Typically, a filter is required to be binary so that a compressive measurement provides the sum of a subset of Raman intensities. If multiple binary filters are used, they are ideally also orthogonal, which means that a certain wavenumber is admitted by at most one filter. Orthogonal filters allow multiple compressive measurements to be taken simultaneously. Whereas a limited number of compressive measurements are not sufficient to reconstruct the complete Raman spectrum without error, they contain enough information to determine the composition of a mixture of chemicals with high accuracy. Seminal work by the groups of Ben-Amotz,^25,26^ Rigneault,^27,28^ and Galland^29−31^ has established a mathematically rigorous procedure to find optimal filters for compressive Raman regression and classification in the presence of photon counting and measurement noise. This procedure requires the noise-free Raman spectra of individual molecular species to be known and assumes specific distributions of the noise. It is therefore not directly applicable to cell products, for which noise-free reference spectra are impossible to obtain and biological variability has a much bigger influence than photon counting or measurement noise.

**Figure 1:**
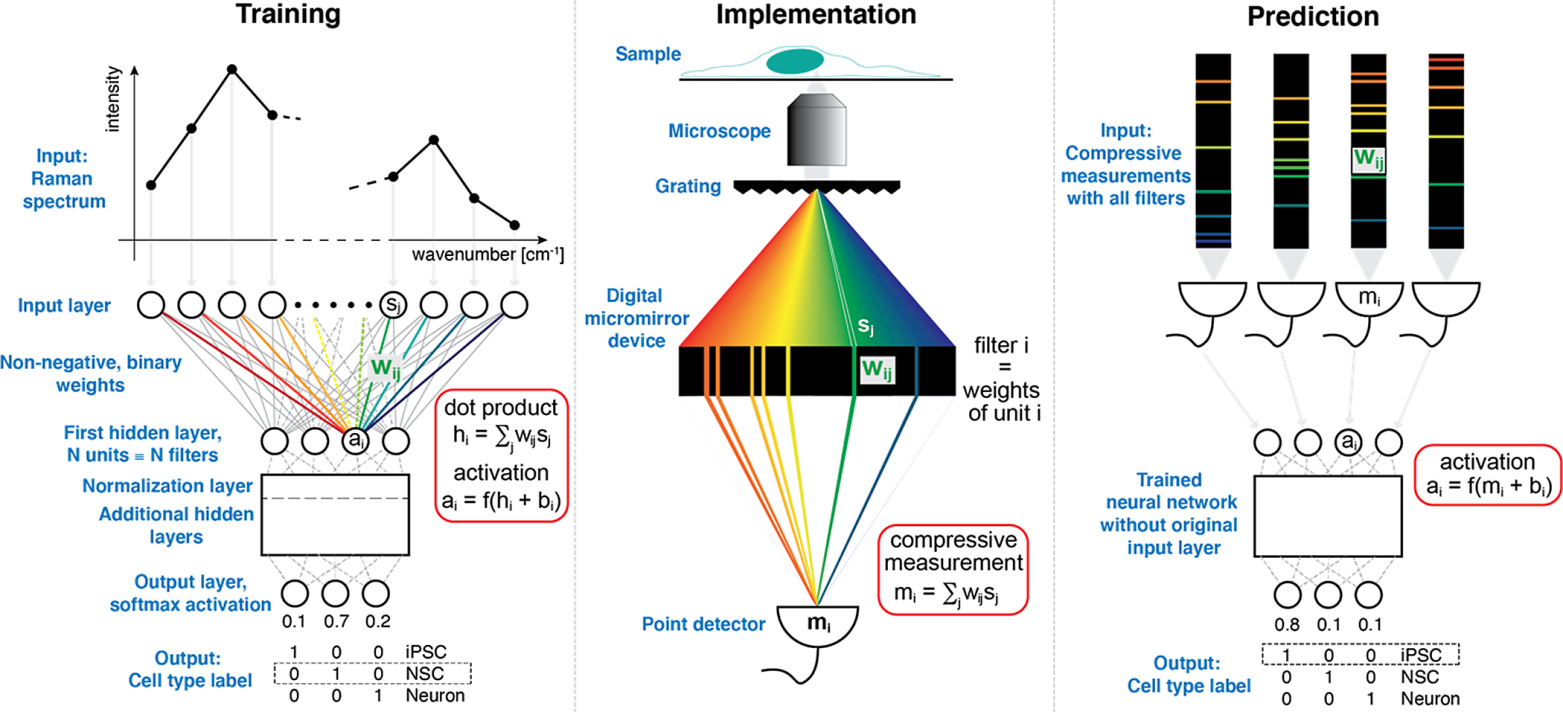
Neural network approach to designing optimal filters for compressive Raman classification of cells. In the training phase, a multilayer perceptron is trained to classify labeled Raman spectra. Calculating the activations *a_i_* of unit *i* in the first hidden layer involves a dot product *h_i_* between the weights **w**_*i*_ of unit *i* and a Raman spectrum **s**. That operation is mathematically equivalent to a compressive measurement. To be usable as optical filters, the weights must be constrained to be non-negative and binary. A normalization layer directly after the first hidden layer ensures efficient training. In the implementation phase, the weights of the *N* units in the first hidden layer of the trained neural network are implemented as *N* optical filters using a suitable optical device. The output *m_i_* of filter *i*, collected by a point detector, is a compressive measurement of a Raman spectrum. For prediction of unseen cells, the measurement *m_i_* replaces the dot product h_i_ in the first hidden layer of the trained neural network.

To extend the compressive Raman approach from the classification of molecular species to the classification of cells, we sought to develop a data-driven approach for the design of optimal filters. Neural network models have achieved impressive accuracy in various classification tasks^32^ and were therefore the natural choice for the problem at hand. The main idea is to train a multilayer perceptron, a simple neural network, on the Raman spectra of cells for which the cell state or type is provided as the label to be learned (Figure 1). In other words, the input to the network is a Raman spectrum and the output is a cell type. Calculating the activation of a unit in the first hidden layer of such a network thus involves the dot product of a Raman spectrum and the weights of that unit. These weights therefore directly correspond to a filter that can be used in compressive sensing. Once the weights are learned in the training phase, they can be implemented with a suitable optical element such as a digital micromirror device. In the prediction phase, compressive Raman measurements are carried out using the filters (i.e., weights) optimized for a specific classification task. The results of these measurements are then used directly instead of the dot products of weights and spectra in the activation function of the first hidden layer. In this manuscript we are concerned with the training phase and demonstrate how optimal filters can be obtained, overcoming two significant problems. First, input data for deep learning models is usually normalized to enable efficient learning. In our compressive sensing scheme, the inputs to the optimized filters are raw, unnormalized Raman spectra. Therefore, we introduced a normalization layer right after the first hidden layer and tested two different kinds of normalization. Second, the weights in a neural network are by default unconstrained, which means they can take arbitrary, continuous values, which might be negative. Such weights are difficult to implement with an optical device. Hence, we developed an approach to obtain binary weights while maintaining high classification accuracy. We tested this optimized neural network model on a data set containing the Raman spectra of induced pluripotent stem cells (iPSCs), neural stem cells (NSCs), and neurons. We were able to achieve up to 86% classification accuracy with only 4 filters and up to 90% with 5 filters. This is comparable to the accuracy of a support vector machine or neural network trained using complete Raman spectra with more than 400 intensities. We also demonstrated that our approach can distinguish naïve and activated T cells with high accuracy using 5 or even fewer filters. Our method thus reduces measurement time by 2 orders of magnitude and thereby enables high-throughput characterization of cell products.

## Results

**Figure 2:**
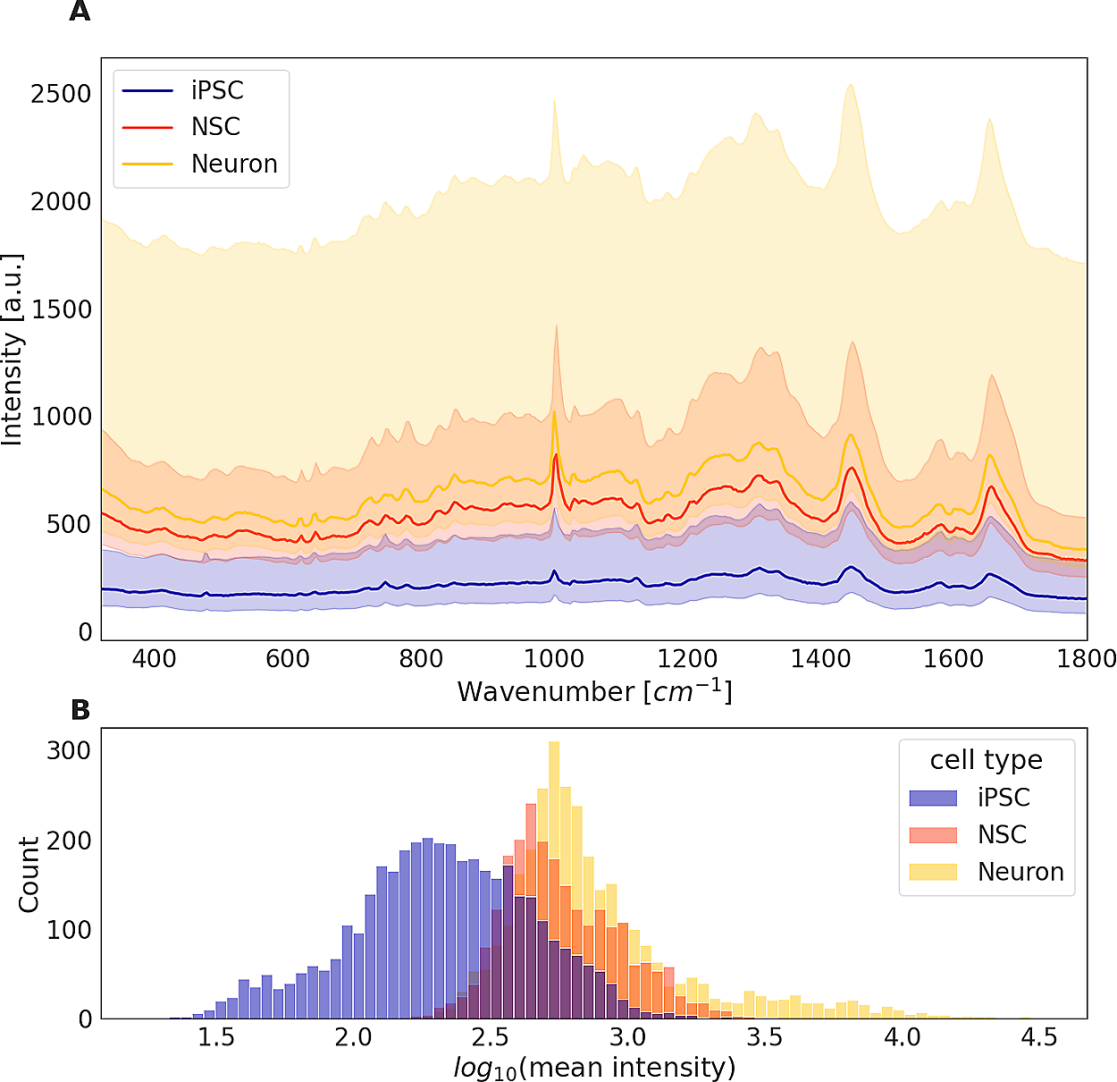
Raw Raman spectra of iPSCs, NSCs, and neurons are strongly overlapping. (A) Raman intensities by cell type before preprocessing. The data set contains 3850 spectra from iPSCs, 2342 from NSCs, and 3116 from neurons. The solid lines show the median per cell type for each wavenumber. The error bands indicate mean absolute deviations calculated separately for positive and negative deviations. (B) Mean intensities of individual Raman spectra before preprocessing.

We first set out to explore the usefulness of Raman spectroscopy for cell classification. Hsu et al.^33^ differentiated iPSCs into NSCs and neurons and measured Raman spectra in individual cells. Following Hsu et al., we only studied the Raman intensities in a “fingerprint region” between 320 and 1800 cm^–1^. Since the raw spectra are strongly overlapping between the different cell types (Figure 2) we adopted standard preprocessing steps to remove a slowly varying baseline and normalize the spectra (Figure S1). Preprocessing reduced, but not eliminated, the overlap of the spectra (Figure S2). Given that the average spectra of different cell types were quite similar (0.97 mean pairwise Pearson correlation), one might expect the classification of individual measurements to be hard. On the contrary, both a support vector machine (SVM) and a multilayer perceptron, a simple neural network (NN), were able to classify the cells with 91 and 90% accuracy, respectively (Figure S3). Most of the misclassifications were due to the confusion of NSCs and neurons (Figure S3A), which might indicate that these cell types are biochemically more similar to each other than to iPSCs. This is supported by low-dimensional embeddings of the Raman spectra (Figure S3B), where iPSCs are largely separate from the other cell types. Most misclassifications occur where spectra from different cell types are close to each other in the embedding space. In summary, properly preprocessed, complete Raman spectra can be easily classified as belonging to different cell types using simple supervised learning methods.

Next, we wanted to establish how much information from a Raman spectrum is really needed to achieve high classification accuracy. We first restricted the input data to subsets of Raman intensities, either by choosing them randomly or picking the intensities with the largest variability across cell types (Figure S4). SVM and NN showed very similar performance, which declined with a reduction in the number of data points used for learning (Figure S5). When points were chosen randomly, accuracy was overall lower and declined more rapidly with the number of used intensities. For comparison, we also tested a simple binning scheme, where intensities were averaged within equally sized intervals. Surprisingly, training on binned spectra resulted in superior performance, compared to using the most variable intensities, from 10 bins upward and only slightly smaller accuracy below 10 bins. Averaging within intervals likely mitigates technical noise corrupting individual intensities. Taken together, we established that feature selection and averaging both have a positive impact on classification performance. Compressive sensing with optimal filters essentially combines both of these aspects and should therefore be able to achieve accurate classification.

We reasoned that an NN would be the most convenient model to use for compressive sensing as it allows us to do feature selection (i.e., the design of optimal filters) and train the downstream classification model at the same time. Calculating the activation of a unit in the first hidden layer of the NN involves computing the dot product of the input (a Raman spectrum) and the weights of the unit. That is mathematically equivalent to taking a compressive Raman measurement with an actual optical filter. This observation is the basis for the suggested approach, which consists of 3 phases (Figure 1). In the training phase, we optimize the weights of an NN classification model using complete, raw Raman spectra labeled with cell types or states. In the implementation phase, we would create separate optical filters for each unit in the first hidden layer. The spectral response of each filter is given by the weights of the corresponding unit since each individual weight is multiplied with a specific intensity in an input Raman spectrum. The prediction phase would consist of compressive Raman measurements, which replace the dot products of spectra and weights in the original NN model. Via the remaining layers of the NN, each measurement, which must include all identified filters, would then lead to a prediction of a cell’s label.

**Figure 3:**
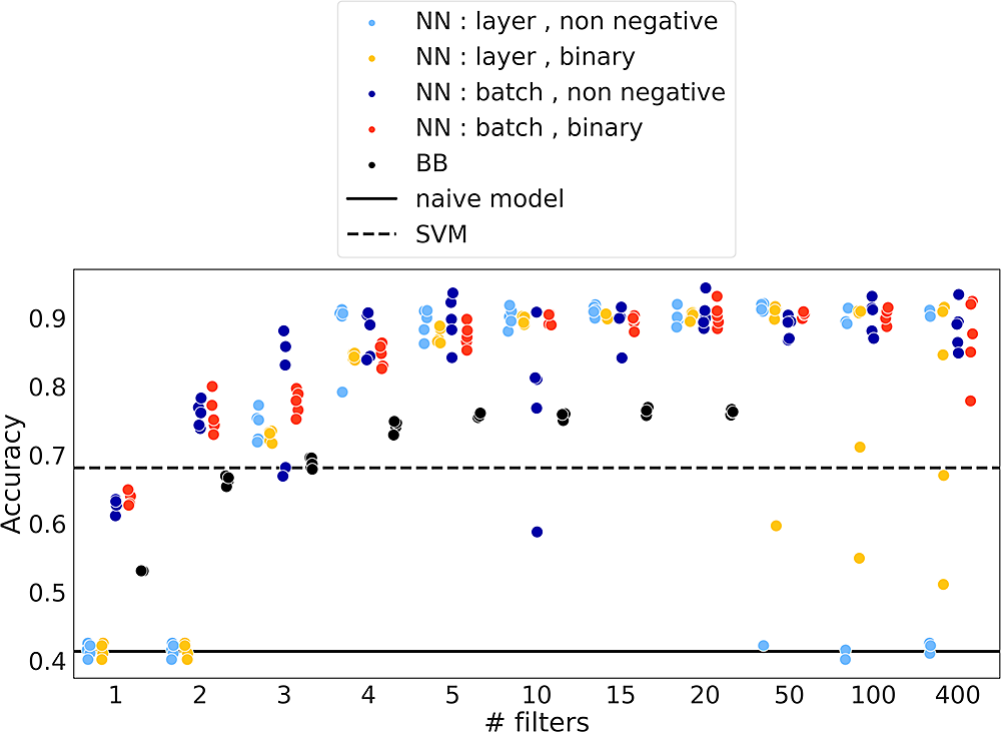
As little as 4–5 compressive measurements are sufficient to classify cell types with high accuracy. Accuracy on held-out test sets for a neural network (NN) model with different numbers of units in the first hidden layer of the NN (= # filters) or the Bhattacharyya bound-based (BB) optimization with different numbers of filters. Two different constraints on the weights in the first hidden layer were used (either “non-negative” or “binary”) as well as two types of normalization (“layer” or “batch” normalization) after the first hidden layer. The 5 data points shown for each choice of parameters (# filters, constraint, normalization) correspond to 5 different splits of the data into training and test set for the NN model. For the BB model, the optimization was run 5 times on the complete data set. The dashed horizontal line indicates the accuracy of a support vector machine trained on raw, unnormalized Raman spectra and the solid horizontal line corresponds to a naïve model that always predicts the most frequent class.

As the compressive measurements will be used as inputs to the NN model, we cannot use any preprocessing based on knowledge of complete spectra (as in Figure S1). However, efficient learning of an NN model typically requires normalized data. Hence, we included a normalization layer after the first hidden layer of the NN and tested two common normalization strategies: batch normalization and layer normalization. Another complication arises from the fact that binary optical filters are easier to implement than filters with continuously varying spectral responses. Consequently, we wanted to add the constraint that the weights of the first hidden layer are binary. Empirically we found that training an NN directly with such a constraint is usually unsuccessful. Instead, we first trained an NN requiring non-negative weights in the first hidden layer. Starting from this pretrained network we then introduced the binarity constraint. Finally, we wanted to study how many optimal filters (i.e., units in the first hidden layer) are necessary to achieve acceptable classification accuracy. Overall, layer normalization gave better results than batch normalization for 4 or more filters (Figure 3). Interestingly, for 5 or more filters, model performance was not reduced by the binarity constraint, if layer normalization was used. The model with binary weights likely benefited from the additional training epochs (starting from the pretrained network with non-negative weights) and the binarity constraint might also effectively regularize the network. Model performance increased with the number of filters up to 50 filters after which it declined and became more variable. Possibly, using more filters introduces additional noise which might outweigh any advantage if classification accuracy is already high. Also, the capacity of the NN increases with the number of units in the first hidden layer, which might lead to a higher variance of the model and therefore worse generalization. Most importantly, using layer normalization and binary weights in the first hidden layer, only 4 filters were necessary to achieve up to 86% classification accuracy and 5 filters resulted in up to 90% accuracy. The model thus achieves a similar accuracy to the models using all 443 Raman intensities (Figure S3). Notably, even with only 3 filters, the NN model is more accurate than an SVM trained on complete, raw Raman spectra. All in all, our results indicate that only 4 to 5 compressive measurements with optimized filters should be sufficient for accurate classification. This would reduce the measurement time by 2 orders of magnitude compared to the acquisition of complete Raman spectra.

Next, we wanted to compare our NN model to the current state-of-the-art filter optimization method, which is based on minimizing an upper bound of the maximum likelihood classification (MLC) error, the Bhattacharyya bound (BB).^29,31^ We first simulated Raman spectra with various levels of correlation assuming photon counting noise as the only source of variability (Figure S6AB). We adopted an optimization algorithm described by Réfrégier et al.^29,31^ and confirmed that the achieved classification error was below the BB (Figure S6C). Across simulated spectra with various levels of correlation, our NN model was comparable in performance to MLC with filters optimized by minimizing the BB (Figure S6D). Since the published filter optimization algorithm assumes multinomially distributed noise, it produced a very poor classification accuracy of around 0.4 when applied to the measured Raman spectra of cells. We hence extended the BB-based optimization algorithm by assuming normally distributed filter outputs and estimating the parameters of the multivariate normal distribution from the data. The MLC accuracy of the resulting filters was markedly increased but was still outperformed by our NN model (Figure 3).

**Figure 4:**
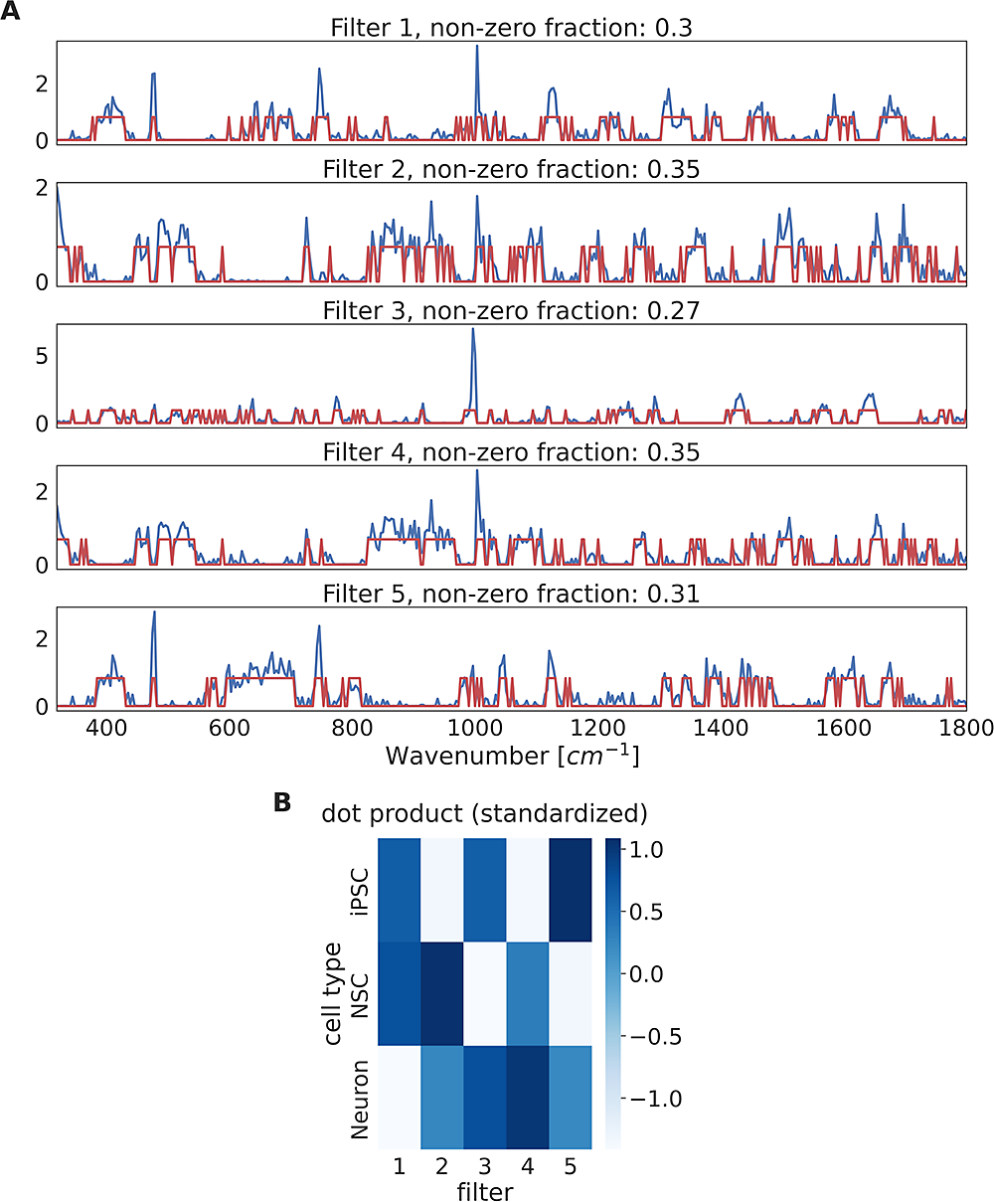
Output patterns of 5 optimal filters encode the cell types. Example of a model with 5 units in the first hidden layer (i.e., 5 filters). (A) Weights learned by the model under the constraint to be non-negative (blue) or binary (red). The fraction of nonzero weights was calculated for the binary filters. (B) Dot products of the 5 filters and raw Raman spectra averaged per cell type and subsequently standardized filter- and cell type-wise.

Since using 10 instead of 5 filters in the NN model improved accuracy only by another 3%, we considered the 5 filter model the optimal trade-off between accuracy and the number of necessary filters. Hence, we decided to further characterize that model (Figure 4) and compare different training scenarios. Inspection of the weights of this model showed that the binary weights closely follow the non-negative weights of the pretrained model (Figure 4A). That might explain why there is usually no decline in accuracy when the binarity constraint is imposed. Inspection of the dot products of spectra and weights of the 5 filters showed that each cell type activated a different subset of filters (Figure 4B). That effectively defines a simple encoding of the 3 cell types. Confusion was biggest between neurons and NSCs (Figure S7A), as for the models based on complete spectra (Figure S3A). Next, we wanted to study how far biological variability across biological replicates or cell lines could impair classification performance. We therefore trained a model with 5 filters on two biological replicates or two cell lines and predicted cells in the third biological replicate or third cell line, respectively (Figure S7B–D). Compared to using all data, accuracy decreased, and confusion increased, as to be expected. Training using two replicates and predicting the third, using cells from all cell lines (resulting in 3 different train-test splits), achieved accuracies of 0.73, 0.79, and 0.87. Training the model on individual cell lines, again training on two biological replicates, and predicting the third (9 different train-test splits) achieved accuracies between 0.66 and 0.9 (mean: 0.79, standard deviation: 0.08). Training on multiple cell lines thus seemed to be somewhat advantageous, likely due to improved generalization. Remarkably, for training across biological replicates, confusion was only appreciably reduced between highly similar cell types (NSCs and neurons), while the model could still distinguish well between iPSCs and the differentiated cell types (Figure S7B,C). Training across cell lines (i.e., using 2 cell lines for training and predicting the third) achieved the lowest accuracies (0.65, 0.81, and 0.67) and highest confusion (Figure S7D). All in all, we observed that classification across biological replicates or cell lines is more challenging, but accuracy can be improved by using more comprehensive training data, as to be expected. We also wanted to test, if the spectral resolution of the optical filter device would have a large influence on classification accuracy. To that end, we trained NN models with 5 filters on measured spectra averaged within equally sized intervals. Surprisingly, accuracy declined only slightly with increased bin size and was still >80% when only 44 wavenumber bins were used for training. The NN model thus produces useful filters even if the spectral resolution of the filters is substantially reduced.

Finally, we were wondering whether our approach is able to classify cell states that are more similar to each other than the cell types in the neuron differentiation system studied above. One might argue that iPSCs and neurons are very different cell types and therefore easy to distinguish by their Raman spectra. As a more challenging system to test our approach in we chose T cell activation, which has high relevance for various applications in immuno-oncology. To that end, we obtained Raman spectra from recent studies in which naïve CD4 T cells or T cells purified from blood were activated for 24 or 72 h, respectively.^34,35^ The median Raman spectra of the two cell states (untreated/activated) were highly similar and the distributions around the median intensities were strongly overlapping (Figures S8AB and S9AB). After training in the same way as described above, NN models with 5 filters achieved accuracies of up to 93% and 89%, respectively (Figures S8C and S9C). Interestingly, for one of the data sets,^35^ layer normalization performed better than batch normalization, in contrast to all other data sets. This might be related to the fact that this data set consisted of only 219 spectra. For both T cell data sets, accuracies above 90% were already achieved with only 3 filters, in contrast to the iPSC differentiation data (Figure 3). This is likely due to the fact that only 2 instead of 3 classes have to be distinguished for the T cell activation system. Overall, the results clearly show that our approach can distinguish highly similar cell states that are relevant for cell therapy applications.

## Discussion

Here we described a neural network approach to the design of optimal filters for compressive Raman classification. Our contribution is distinct from seminal prior work^25−31^ in several ways. First, it does not require noise-free reference spectra or assume a specific noise distribution but learns filters directly from labeled experimental data using a neural network. Deep learning has been used extensively in connection with compressive sensing,^36^ but most applications are in signal reconstruction.^37−39^ Calderbank et al.^40^ introduced the concept of compressed learning, which uses compressed measurements directly as input, for example to a classifier, without reconstruction of the signal. Similar to schemes for compressed learning on images,^41,42^ which simultaneously learn a sensing matrix and an image classifier, we simultaneously learn a set of spectral filters and a spectrum classifier. Additionally, we introduced specific constraints on the first hidden layer such that the learned weights can be implemented directly by physical optical filters.

The method was tested in a neuron differentiation data set comprising three different cell types, collected by Hsu et al.,^33^ as well as T cell activation studies,^34,35^ which reported Raman spectra of untreated and activated T cells. We demonstrated that the smallest NN model that delivered high classification accuracy (>90%) in the neuron differentiation system required only 5 filters. For the T-cell activation experiments, which contain only 2 cell states, high accuracies were already achieved with only 3 filters. The number of filters necessary to distinguish a certain number of cell types depends on multiple experimental factors, such as measurement noise, biological variability between individual cells, and the difference in chemical composition between the cell types. We speculate that up to 10 cell types could be distinguishable with 10–20 filters. Using more filters than that would defy the purpose of our approach, which is to speed up Raman classification (see our estimation of measurement time reduction below).

As to be expected, our model confused more similar cell types more frequently. Hsu et al. reported that, after preprocessing and t-distributed stochastic neighbor (t-SNE) embedding of the spectra, a stack of multiple classifiers can be 97.5% accurate. The lower accuracy of our model is to be expected for multiple reasons. First, we cannot use preprocessing or t-SNE embedding since both operations require knowledge of the complete spectra, which are not obtained with compressive measurements. Second, since we opted to use a neural network for simultaneous filter design and training of a classification model, we only trained a single classifier. In fact, single classifiers without t-SNE embedding tested by Hsu et al. were at best 92.8% accurate, which is comparable to the performance of our model. To improve accuracy further, information about the baseline could be included, but that would require additional filters that can predict baselines from raw spectra. Having found that a simple binning scheme improved classification performance (Figure S5), we expect that a reduction of experimental noise by averaging would be beneficial. Combining multiple point measurements of a single cell or collecting signal from a larger volume might therefore be possibilities to further increase classification performance. In principle, it could be a concern that the NN model produces filters with very low optical efficiency, i.e., a small amount of nonzero elements. In practice, we found that roughly 1/3 of filter elements are nonzero, which should give a reasonable optical efficiency. It is important to note that our method is trained on actual experimental data, which contains all expectable variability. Thus, even filters with low optical efficiency should perform well in practice. If desired, an additional loss term that penalizes zero filter elements could be added easily to the model definition.

The optimal filters inferred by our method are meant to be implemented using a physical optical filter (hence the binarity constraint, which simplifies the experimental implementation). The schematic in Figure S10A shows a possible realization of a compressive Raman spectrometer. After the optimal filters are learned, using full spectra and associated sample labels, only compressive measurements will be taken. The compression (or filtering) would happen in the optical device. No further filtering would be applied during data analysis, but the classification component of the neural network (i.e., the network with the input layer removed) would be used to classify the samples. Figure S10B summarizes the envisioned workflow of spectrometer calibration and routine measurements. It remains to be shown empirically that this idea works in practice.

To estimate the potential reduction in measurement time resulting from our scheme, we can consider the reported experimental parameters.^33^ Each of the Raman spectra measured by Hsu et al. comprised 1021 wavenumbers and took 5 s to acquire with an HR Evolution confocal Raman microscope (Horiba), which uses a CCD chip to image the spectrum. To make our results comparable to the original publication, we restricted our analysis to a “fingerprint region” comprising 443 wavenumbers, but in principle, we could have used all 1021 wavenumbers. The optimal filters identified by the neural network have an optical efficiency (i.e., fraction of transmitted wavenumbers) of approximately 1/3. That means that the expected output of a filter is 1021/3 = 340 times the signal from a single wavenumber in a complete spectrum. That means that the acquisition time can be reduced by a factor of 340. However, since we have to use 5 filters, the reduction in total measurement time is approximately 340/5 = 68. Given that single-point detectors typically have much lower noise than CCD chips, the acquisition time can likely be lowered even further which warrants the statement that measurement time can be reduced by 2 orders of magnitude. A switchable optical filter can be implemented by a digital micromirror device (DMD); see Figure S10A. The switching time of a DMD is in the submillisecond regime and therefore negligible. The performance of our method could be improved even further by using orthogonal filters. In a set of orthogonal filters, a certain wavenumber is transmitted by exactly one filter. For such a set of filters, the DMD could be used to send the signal from all filters simultaneously to multiple single-point detectors.

We envision two conceptually different ways in which our method could be used in practice: longitudinal experiments where multiple states of a single cell line are classified repeatedly or classification of states in a cell line that was not used for training. As demonstrated (Figure S7), our method will likely work better in the first application. We suspect that changes over time, due to variability in handling or slow drift with passage number, could be detectable as increasing uncertainty of the neural network prediction. In this case, the user could be prompted to retrain the classifier. Uncertainty can be estimated using ensemble techniques or Bayesian approaches,^43^ which are beyond the scope of the current manuscript. The second application is certainly more challenging, which is reflected by the reduced performance when trying to predict cell types for a cell line that was not included in the training set (Figure S7D).

In both applications, strong batch effects, due to technical or biological factors, could be problematic, but there are multiple conceivable ways to reduce their impact. First, currently available data sets contain only modest numbers of biological replicates or cell lines. As robotics and automated cell culture become more commonplace, at least in an industrial setting, technical variability will decline, and it will be much easier to obtain Raman spectra from dozens of cell lines. We expect the performance on a held-out cell line to improve with the number of cell lines in the training set, as it becomes more likely that cell lines similar to the test cell line have been seen by the model during training. Second, cell lines, culture conditions, etc., can be considered nuisance parameters that could be provided during training to increase robustness. For example, Lotfollahi et al. have recently used a conditional variational autoencoder to build a reference cell type atlas despite significant variation between the data sets used for training.^44^ They provided data set labels as input to the autoencoder. A similar approach might help our Raman classification model generalize to unseen cell lines. Finally, we can envision some form of adversarial learning where the model is tasked to predict both batch and cell type, but the loss function penalizes correct batch prediction. As the amounts of data necessary to implement such sophisticated ideas are currently unavailable, our manuscript should be seen as a proof-of-concept to be improved further in the future.

In this study, we demonstrated our approach in two different biological systems, which highlights its broad applicability. A model trained on iPSC differentiation data could be useful to assess proper cell type specification prior to the application of differentiated cells in a clinical setting. Likewise, the model could be trained to assess the reprogramming of somatic cells to iPSCs. The classification of T cells and other immune cell states might be very useful in various immuno-oncology applications. In conclusion, we have demonstrated a possible way to leverage compressive Raman measurements for the classification of cells, which could have wide-ranging applicability in the manufacturing of cell-based therapies. We intend to demonstrate the experimental implementation of optimal filters and the use of compressive Raman measurements in a future study.

## Methods

### Data Sets and Preprocessing

We downloaded the Raman spectra collected by Hsu et al.^33^ from https://osf.io/9env5/. After removing failed measurements (spectra with only zeros), we retained 9308 spectra: 3850 spectra from 180 iPSCs, 2342 spectra from 176 NSCs, and 3116 spectra from 180 neurons. On average, 17.4 measurements were taken per cell. Measurements are distributed across 3 cell lines and 3 technical replicates per cell line. For each technical replicate in each cell line, 20 cells were measured, with one exception, where data for only 16 cells were reported. A more detailed breakdown of the data set is given in Table S1. An overview of the raw spectra is shown in Figure 2. The Raman spectra of the T cell activation experiments^34,35^ were obtained directly from their creators. An overview of the raw spectra is shown in supplementary Figures S8A and S9A. The breakdowns of these data sets can be found in supplementary Tables S2 and S3.

For the results shown in Figures S3 and S5, spectra were preprocessed by baseline removal and normalization prior to classification with the support vector machine and neural network model A (see below). A slowly varying baseline was estimated by asymmetric least-squares smoothing, which was introduced by EIlers et al.^45^ and applied and further developed in the context of Raman spectra by He et al.^46^ For each spectrum we ran the smoothing algorithm for 10 iterations with parameters reported in the literature^47^ (smoothness penalty parameter λ of 10,^6^ asymmetry parameter *p* of 0.1) and subtracted the resulting baseline from the raw spectrum. Baseline-corrected spectra were subsequently normalized by dividing by the sum of all intensities. A preprocessing example is shown in Figure S1. Figure S2 shows an overview of the preprocessed spectra.

### Support Vector Machine (SVM) Classification

For SVM classification, we used the *SVC* method from the Python package *Scikit-learn* (version 1.1.1) with default parameters. By default, *SVC* uses a radial basis function (or squared-exponential) kernel, and the length scale of the kernel is determined by this formula: 1/(number of features * variance of flattened training data matrix). 20% of the data set was held out for testing, using the *train_test_split* function from *Scikit-learn*. Prediction accuracy was determined on the test set using the *accuracy_score* function form *Scikit-learn*. For the classification of preprocessed spectra, the *StandardScaler* function of *Scikit-learn* was used to standardize the training data feature-wise (i.e., per wavenumber). Raw spectra were not normalized prior to SVM classification.

### Neural Network (NN) Models

The Python package *tensorflow* (version 2.8.0) was used to build, train, and test all NN models. Two different NN models, termed A and B were used for the prediction of cell type labels from preprocessed spectra or raw spectra, respectively. Model A, which was used for preprocessed spectra, consisted of 3 fully connected layers: an input layer with 443 units, where each unit corresponds to a wavenumber, a hidden layer with 10 units and ReLU activation function, and an output layer with 3 units and softmax activation function (Figure S11A). Layer weights were initialized using the default initializer (Glorot uniform initializer). Model B, which was used for raw Raman spectra, consisted of 5 layers: an input layer where each unit corresponds to a wavenumber; a hidden layer with N units and ReLU activation function, where each unit corresponds to a filter to be optimized; a normalization layer, which performs either batch or layer normalization; a hidden layer with 10 units and ReLU activation function and an output layer with 3 units and softmax activation function (Figure S11B). For the data from Hsu et al., Pavillon et al., and Chaudhary et al. the input layer had 443, 1024, and 1397 units, respectively. The weights of layers 1 (input), and 5 (output) were initialized with the Glorot uniform initializer. The beta and gamma parameters of the normalization layer were initialized to zero and one, respectively. The weights of layer 2 (first hidden layer) and layer 4 (second hidden layer) were initialized using He Uniform initialization. To impose the non-negativity constraint on the weights of the first layer we used the *NonNeg* constraint from *tensorflow*. For the binarity constraint, we developed a custom constraint method:
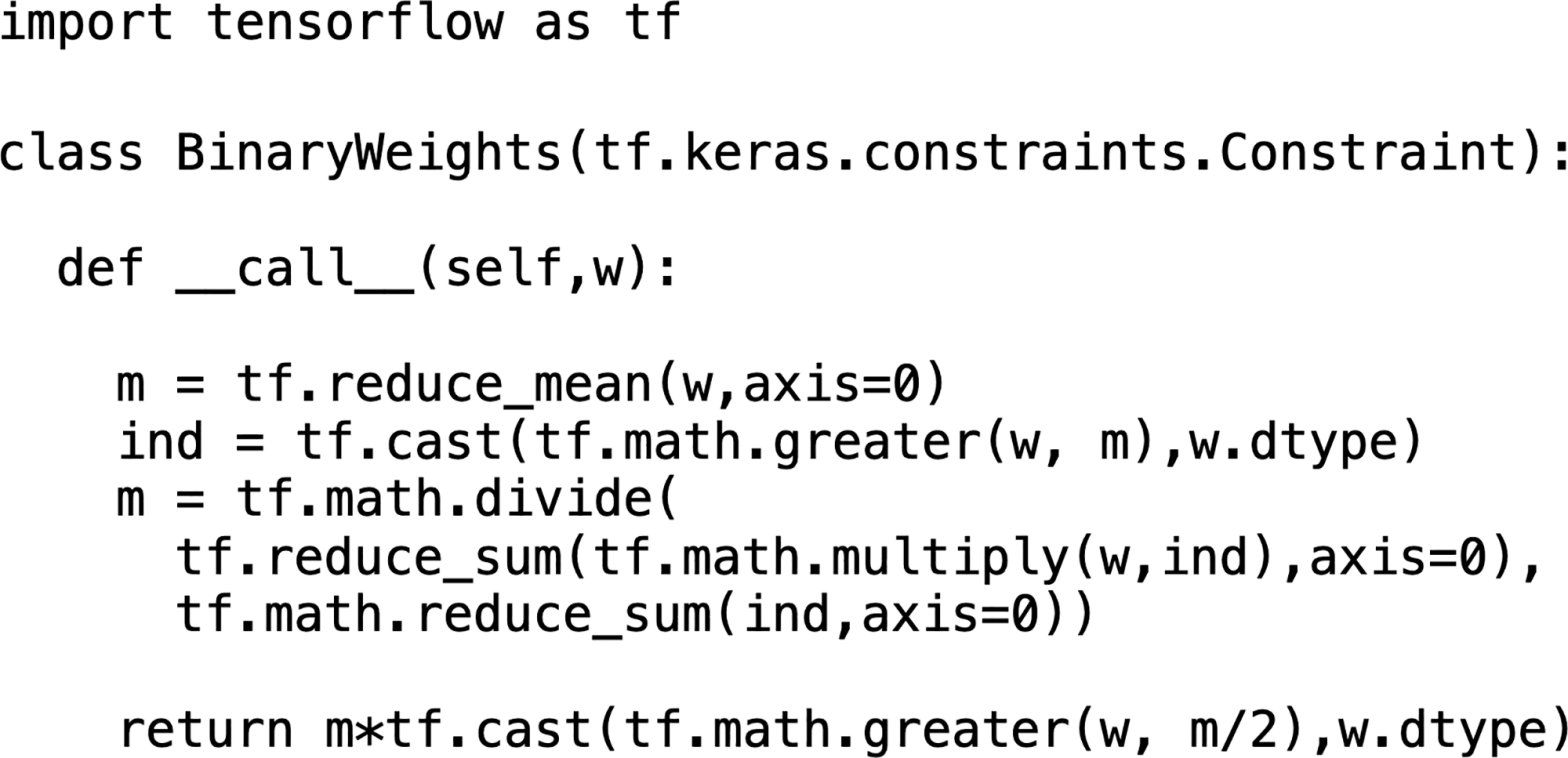


This method first determines all weights that are larger than the mean weight and calculates a new mean of just those “high” weights. Then, all weights exceeding half the mean of the high weights are set to that mean, and all other weights are set to 0. This method ensures binary filter elements, but the nonzero elements are not necessarily 1. As an arbitrary scaling factor can be easily absorbed into the downstream classification model, the optical filters can be implemented with elements restricted to 0 and 1.

Prior to training, a held-out test set consisting of 20% of the data was created with the *train_test_split* function from *Scikit-learn* using stratification by classes (i.e., cell types). In the case of the preprocessed Raman spectra, the training data was standardized feature-wise (i.e., per wavenumber) using the *StandardScaler* function of *Scikit-learn* prior to training model A for 20 epochs with a batch size of 32. In the case of the raw spectra, there was no normalization prior to training. Model B was trained on the raw spectra for 200 epochs with a batch size of 16. For the simulated spectra, 20 epochs and a batch size of 128 were used.

To obtain a model with binary weights in the first hidden layer, model B was first trained for 200 epochs with a learning rate of 0.01 and a non-negativity constraint on the weights in the first hidden layer. The weights of the resulting model were then copied to a new network with identical architecture but a binarity constraint on the weights in the first hidden layer. Training of this new model on the same train-test split for another 200 epochs and with a learning rate of 0.001 resulted in the final model with binary weights in the first hidden layer. Initially, we used 0.01 as the learning rate for both training steps but found that decreasing the learning rate to 0.001 during training with binarity constraint increased accuracy, in particular for the T cell activation data sets. 0.001 is the default learning rate for the stochastic gradient descent optimizer in *tensorflow*. Sparse categorical cross entropy was used as the loss function in all cases and all models were trained using stochastic gradient descent. To monitor the effect of batch size and number of epochs, we used a validation set during training. For the number of epochs and batch sizes reported, validation accuracy was stable and similar to training accuracy, which argues against overfitting.

For each of the 5 different train–test splits, the model was trained multiple times varying all relevant hyperparameters (number of units N in the first hidden layer, type of normalization in the normalization layer, and constraint on the weights in the first hidden layer), which made the results comparable across hyperparameters. 20% of the training set was held out as a validation set, using the *train_test_split* function from *Scikit-learn*, and the model was trained on the remaining 64% of the complete data set. For each train-test split, the model was trained 3 times (with different, bootstrapped validation sets) and the model with the best performance on a validation set was selected for evaluation on the test set. Performance on the test set is reported in Figure 3 or Figures S8C and S9C. For the neuron differentiation data set, we studied 44 different models in total: 11 choices for the number of units in the first hidden layer, 2 types of normalization (batch or layer), and 2 different constraints on the first hidden layer (see Figure 3). For the T cell activation data sets we only considered 6 choices for the number of units in the first hidden layer, resulting in 24 different models (see Figures S8C and S9C). For the classification of simulated data (see the next section), *N* = 3 units in the first hidden layer were used.

### Simulations

Raman spectra were simulated following an established approach.^29,31^ Intensities for *K* = 50 wavenumbers *k* were drawn from an exponential probability density with unit mean, raised to the power α = 3, and subsequently divided by the sum of all intensities, for normalization.

To create spectra **s**_*j*_ for *M* = 3 molecular species with controllable levels of correlation, we first simulated *M* + 1 spectra **r**_*j*_ and defined spectrum *M* + 1 as the “common” spectrum. To create correlated spectra **s**_*j*_, linear combinations of the spectra **r**_*j*_ were calculated:


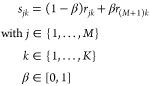
1

For β = 1, all 3 spectra are identical to the common spectrum, for β = 0 the spectra are independent random variables (see Figure S6A,B).

To simulate photon counting noise on a spectrum, photon numbers **ν** were drawn randomly from a multinomial distribution with the distribution parameters given by the normalized spectral intensities *s_jk_* and a total number of photons *N*_phot_:
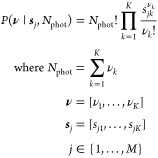
2

For training and testing the neural network model, 10,000 samples were drawn from this distribution for each molecular species, where each sample is a complete spectrum with noise. The split into training and test sets as well as the hyperparameters used for training the NN are described in the previous section. Since we simulated 3 molecular species, the first hidden layer was chosen to contain 3 units.

To simulate counting noise after a filter, first, the filter outputs μ_*ij*_ were calculated as the dot product between a filter **F**_*i*_ and a spectrum **s**_*j*_:
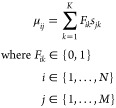
3

Since the spectra are normalized and each filter element is binary (either 0 or 1), μ_*ij*_ is the fraction of transmitted signal intensity, i.e., the optical efficiency, of filter *i* for molecular species *j*. To simulate photon counting noise on a filter output, photon numbers **n** were drawn randomly from a multinomial distribution with the distribution parameters given by the normalized filter outputs *p_ij_* and a total number of photons *N*_phot_:
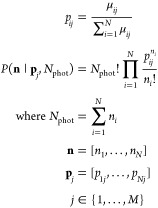
4

Note that it is entirely equivalent to introduce photon counting noise before or after a filter since a linear combination of multinomial-distributed random variables is again multinomial-distributed. Since the filters we find by BB-based optimization (see next section) have an approximate optical efficiency of 1%, spectra simulated with *N*_phot_ photons are equivalent to filter outputs simulated with 0.01 *N*_phot_ photons.

### BB-Based Optimization of Filters

Réfrégier et al.^29,31^ developed compressive Raman classification based on the principle of maximum likelihood. In short, a measurement of photon numbers **n** for the output of *N* filters is classified by finding the spectrum that had the biggest likelihood of giving rise to that measurement:
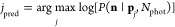
5

The BB is an upper bound of the classification error, which has the following form for multinomial (i.e., photon counting) noise:
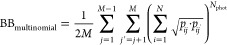
6

This expression is valid, if each class is equally frequent. As shown by Réfrégier et al.,^29,31^ filters can be optimized by minimizing the BB bound. The optimization algorithm starts with a set of random filters and attempts “flips” of filter elements from 0 to 1 or 1 to 0. Such flips are accepted, if they reduce the BB bound. If the only objective is a minimal BB bound, the resulting filters might have only a few nonzero elements, which corresponds to low optical efficiency. Adding a certain optical efficiency as another objective requires makes the optimization more challenging. Instead, here, we initialize each filter with 1/3 of their elements set to 1 and instead of flipping individual elements, we attempt to swap a randomly chosen 0 with a randomly chosen 1 from the same filter, which preserves the fraction of nonzero elements. A swap is accepted if it reduces the BB bound. We found that this algorithm converged quickly and led to filters with approximately 1% optical efficiency. To make a fair comparison between the BB-based optimization and the NN model, we did not require filters to be orthogonal, which would likely reduce the achievable classification accuracy. The actual classification error or accuracy of optimized filters was then obtained by classifying simulated filter outputs, where photon counting noise was introduced at the level of the filters (see previous section).

To apply BB-based optimization to the measured Raman spectra of cells, we had to adapt the scheme developed by Réfrégier et al.^29,31^ In contrast to measurements of simple molecules, the variability between spectra of the same class (i.e., cell type) is not sufficiently described by (multinomial) photon counting noise or other measurement noise with a simple distribution. The BB given above is therefore not appropriate. Instead, we made the assumption that filter outputs are normally distributed since a closed-form expression for the BB exists in that case.^48^ Means **m**_*j*_ and covariance matrices **Σ**_*j*_ of filter outputs were estimated from all the *N*_data,*j*_ spectra of cell type *j* in the data set:
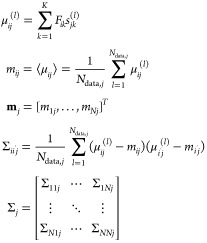
7where the superscript (*l*) indexes the measured samples. In the case of normally distributed noise, the BB is given by^48^
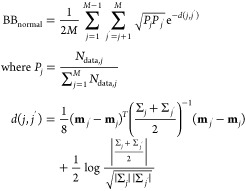
8

As the numbers of spectra from different cell types *N*_data,*j*_ are not equal, this expression contains the probabilities *P_j_* that a random sample from the data set belongs to cell type *j*.

The same optimization algorithm as above was used, albeit with the BB for normally distributed filter outputs. The actual classification error or accuracy of optimized filters was then obtained by maximum likelihood classification of measured spectra:
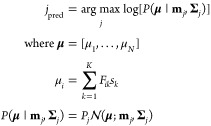
9where **s** is a measured spectrum, **μ** is the corresponding filter output, and *N*(**μ**;**m**_*j*_, **Σ**_*j*_) is a multivariate normal distribution with means **m**_*j*_ and covariance matrix **Σ**_*j*_.

## Data Availability

The data set on iPSC differentiation collected by Hsu et al.^33^ can be downloaded from https://osf.io/9env5/. The T cell activation data set collected by Chaudhary et al.^35^ can be obtained from Zenodo at https://zenodo.org/records/10955443. Both data sets were made publicly available under a Creative Commons Attribution 4.0 International Public License. The data set on T cell differentiation by Pavillon et al.^34^ can be obtained from their creators upon reasonable request. The jupyter notebook used to create all figures in this study is publicly available from GitHub (https://github.com/semraulab/raman).

## Supporting Information Available

Baseline removal and normalization, overlap of spectra with and without preprocessing, support vector machine and neural network classification of spectra, selection of informative Raman intensities by variability across cell types, increase of classification accuracy on preprocessed spectra with included wavenumbers or wavenumber bins, performance of BB-based optimization and neural network model on simulated data, impact of biological variability on prediction accuracy, classification of T cell activation (Pavillon et al., Scientific Reports, 2023), classification of T cell activation (Chaudhary et al., Spectrochimica Acta Part A: Molecular and Biomolecular Spectroscopy, 2023), schematic of a compressive Raman spectrometer and calibration workflow, neural network models, breakdown of data sets created by Hsu et al., breakdown of data sets created by Pavillon et al., and breakdown of data sets created by Chaudhary et al. (PDF)

## References

[ref1] El-Kadiry A. E.-H.; Rafei M.; Shammaa R. Cell Therapy: Types, Regulation, and Clinical Benefits. Frontiers Medicine 2021, 8, 756029 10.3389/fmed.2021.756029. PMC8645794 34881261

[ref2] Andrzejewska A.; Lukomska B.; Janowski M. Concise Review: Mesenchymal Stem Cells: From Roots to Boost. Stem Cells 2019, 37 (7), 855–864. 10.1002/stem.3016. 30977255 PMC6658105

[ref3] Hwang J. J.; Rim Y. A.; Nam Y.; Ju J. H. Recent Developments in Clinical Applications of Mesenchymal Stem Cells in the Treatment of Rheumatoid Arthritis and Osteoarthritis. Front Immunol 2021, 12, 631291 10.3389/fimmu.2021.631291. 33763076 PMC7982594

[ref4] Pittenger M. F.; Discher D. E.; Péault B. M.; Phinney D. G.; Hare J. M.; Caplan A. I. Mesenchymal Stem Cell Perspective: Cell Biology to Clinical Progress. Npj Regen Medicine 2019, 4 (1), 22 10.1038/s41536-019-0083-6. PMC6889290 31815001

[ref5] June C. H.; O’Connor R. S.; Kawalekar O. U.; Ghassemi S.; Milone M. C. CAR T Cell Immunotherapy for Human Cancer. Science 2018, 359 (6382), 1361–1365. 10.1126/science.aar6711. 29567707

[ref6] Mallapaty S. Pioneering Stem-Cell Trials in Japan Report Promising Early Results. Nature 2022, 609, 235 10.1038/d41586-022-02232-7. 36002739

[ref7] Bashor C. J.; Hilton I. B.; Bandukwala H.; Smith D. M.; Veiseh O. Engineering the next Generation of Cell-Based Therapeutics. Nat. Rev. Drug Discov. 2022, 21, 655 10.1038/s41573-022-00476-6. 35637318 PMC9149674

[ref8] Aijaz A.; Li M.; Smith D.; Khong D.; LeBlon C.; Fenton O. S.; Olabisi R. M.; Libutti S.; Tischfield J.; Maus M. V.; Deans R.; Barcia R. N.; Anderson D. G.; Ritz J.; Preti R.; Parekkadan B. Biomanufacturing for Clinically Advanced Cell Therapies. Nat. Biomed. Eng. 2018, 2 (6), 362–376. 10.1038/s41551-018-0246-6. 31011198 PMC6594100

[ref9] Heathman T. R.; Nienow A. W.; McCall M. J.; Coopman K.; Kara B.; Hewitt C. J. The Translation of Cell-Based Therapies: Clinical Landscape and Manufacturing Challenges. Regen. Med. 2015, 10 (1), 49–64. 10.2217/rme.14.73. 25562352

[ref10] Weil B.; Hanley P. J.; Lowdell M. Proposal for the International Society for Cell & Gene Therapy Position Statement on Assays for the Quality Control and Potency Assessment of Adoptive Cellular Immunotherapies. Cytotherapy 2019, 21 (3), 367–375. 10.1016/j.jcyt.2019.02.001. 30890307

[ref11] Abou-el-Enein M.; Elsallab M.; Feldman S. A.; Fesnak A. D.; Heslop H. E.; Marks P.; Till B. G.; Bauer G.; Savoldo B. Scalable Manufacturing of CAR T Cells for Cancer ImmunotherapyClinical Production of CAR T Cells. Blood Cancer Discovery 2021, 2 (5), 408–422. 10.1158/2643-3230.BCD-21-0084. 34568831 PMC8462122

[ref12] Huang S.; Wang X.; Wang Y.; Wang Y.; Fang C.; Wang Y.; Chen S.; Chen R.; Lei T.; Zhang Y.; Xu X.; Li Y. Deciphering and Advancing CAR T-Cell Therapy with Single-Cell Sequencing Technologies. Mol. Cancer 2023, 22 (1), 80 10.1186/s12943-023-01783-1. 37149643 PMC10163813

[ref13] Costa L. A.; Eiro N.; Fraile M.; Gonzalez L. O.; Saá J.; Garcia-Portabella P.; Vega B.; Schneider J.; Vizoso F. J. Functional Heterogeneity of Mesenchymal Stem Cells from Natural Niches to Culture Conditions: Implications for Further Clinical Uses. Cell. Mol. Life Sci. 2021, 78 (2), 447–467. 10.1007/s00018-020-03600-0. 32699947 PMC7375036

[ref14] Odum J. N. The Impact of Process Closure on Biomanufacturing Risk. BioProcess J. 2023, 22, 10.12665/j22oa.odum.

[ref15] Gannon P. O.; Harari A.; Auger A.; Murgues C.; Zangiacomi V.; Rubin O.; Lavoie K. E.; Guillemot L.; Rodrigo B. N.; Nguyen-Ngoc T.; Rusakiewicz S.; Rossier L.; Boudousquié C.; Baumgaertner P.; Zimmermann S.; Trueb L.; Iancu E. M.; Sempoux C.; Demartines N.; Coukos G.; Kandalaft L. E. Development of an Optimized Closed and Semi-Automatic Protocol for Good Manufacturing Practice Manufacturing of Tumor-Infiltrating Lymphocytes in a Hospital Environment. Cytotherapy 2020, 22 (12), 780–791. 10.1016/j.jcyt.2020.07.011. 33069566

[ref16] Waisman A.; Greca A. L.; Möbbs A. M.; Scarafía M. A.; Velazque N. L. S.; Neiman G.; Moro L. N.; Luzzani C.; Sevlever G. E.; Guberman A. S.; Miriuka S. G. Deep Learning Neural Networks Highly Predict Very Early Onset of Pluripotent Stem Cell Differentiation. Stem Cell Rep. 2019, 12 (4), 845–859. 10.1016/j.stemcr.2019.02.004. PMC6449871 30880077

[ref17] Gamal W.; Wu H.; Underwood I.; Jia J.; Smith S.; Bagnaninchi P. O. Impedance-Based Cellular Assays for Regenerative Medicine. Philosophical Transactions Royal Soc. B Biological Sci. 2018, 373 (1750), 20170226 10.1098/rstb.2017.0226. PMC5974449 29786561

[ref18] Croce A. C.; Bottiroli G. Autofluorescence Spectroscopy and Imaging: A Tool for Biomedical Research and Diagnosis. Eur. J. Histochem 2014, 58 (4), 2461 10.4081/ejh.2014.2461. 25578980 PMC4289852

[ref19] Shipp D. W.; Sinjab F.; Notingher I. Raman Spectroscopy: Techniques and Applications in the Life Sciences. Adv. Opt Photonics 2017, 9 (2), 315 10.1364/AOP.9.000315.

[ref20] Ember K. J. I.; Hoeve M. A.; McAughtrie S. L.; Bergholt M. S.; Dwyer B. J.; Stevens M. M.; Faulds K.; Forbes S. J.; Campbell C. J. Raman Spectroscopy and Regenerative Medicine: A Review. Npj Regen Medicine 2017, 2 (1), 12 10.1038/s41536-017-0014-3. PMC5665621 29302348

[ref21] Pettinato G.; Coughlan M. F.; Zhang X.; Chen L.; Khan U.; Glyavina M.; Sheil C. J.; Upputuri P. K.; Zakharov Y. N.; Vitkin E.; D’Assoro A. B.; Fisher R. A.; Itzkan I.; Zhang L.; Qiu L.; Perelman L. T. Spectroscopic Label-Free Microscopy of Changes in Live Cell Chromatin and Biochemical Composition in Transplantable Organoids. Sci. Adv. 2021, 7 (34), eabj2800 10.1126/sciadv.abj2800. 34407934 PMC8373132

[ref22] Candès E. J.; Romberg J.; Tao T. Robust Uncertainty Principles: Exact Signal Reconstruction From Highly Incomplete Frequency Information. IEEE Trans. Inf. Theory 2006, 52 (2), 489–509. 10.1109/TIT.2005.862083.

[ref23] Candes E. J.; Wakin M. B. An Introduction To Compressive Sampling. IEEE Signal Process. Mag. 2008, 25 (2), 21–30. 10.1109/MSP.2007.914731.

[ref24] Foucart S.; Rauhut H.; Foucart S.; Rauhut H.; A Mathematical Introduction to Compressive Sensing. In Applied and Numerical Harmonic Analysis 2013, 10.1007/978-0-8176-4948-7.

[ref25] Wilcox D. S.; Buzzard G. T.; Lucier B. J.; Rehrauer O. G.; Wang P.; Ben-Amotz D. Digital Compressive Chemical Quantitation and Hyperspectral Imaging. Analyst 2013, 138 (17), 4982–4990. 10.1039/c3an00309d. 23817274

[ref26] Wilcox D. S.; Buzzard G. T.; Lucier B. J.; Wang P.; Ben-Amotz D. Photon Level Chemical Classification Using Digital Compressive Detection. Anal. Chim. Acta 2012, 755, 17–27. 10.1016/j.aca.2012.10.005. 23146390

[ref27] Scotté C.; Aguiar H. B. de; Marguet D.; Green E. M.; Bouzy P.; Vergnole S.; Winlove C. P.; Stone N.; Rigneault H. Assessment of Compressive Raman versus Hyperspectral Raman for Microcalcification Chemical Imaging. Anal. Chem. 2018, 90 (12), 7197–7203. 10.1021/acs.analchem.7b05303. 29761698

[ref28] Sturm B.; Soldevila F.; Tajahuerce E.; Gigan S.; Rigneault H.; Aguiar H. B. de. High-Sensitivity High-Speed Compressive Spectrometer for Raman Imaging. ACS Photon. 2019, 6 (6), 1409–1415. 10.1021/acsphotonics.8b01643.

[ref29] Réfrégier P.; Chevallier E.; Galland F. Compressed Raman Classification Method with Upper-Bounded Error Probability. Opt. Lett. 2019, 44 (23), 5836 10.1364/OL.44.005836. 31774792

[ref30] Réfrégier P.; Scotté C.; Aguiar H. B. de; Rigneault H.; Galland F. Precision of Proportion Estimation with Binary Compressed Raman Spectrum. J. Opt. Soc. Am. 2017, 35 (1), 125 10.1364/josaa.35.000125. 29328101

[ref31] Réfrégier P.; Galland F. Bhattacharyya Bound for Raman Spectrum Classification with a Couple of Binary Filters. Opt. Lett. 2019, 44 (9), 2228 10.1364/OL.44.002228. 31042190

[ref32] Emmert-Streib F.; Yang Z.; Feng H.; Tripathi S.; Dehmer M. An Introductory Review of Deep Learning for Prediction Models With Big Data. Frontiers Artif Intell 2020, 3, 4 10.3389/frai.2020.00004. PMC7861305 33733124

[ref33] Hsu C.-C.; Xu J.; Brinkhof B.; Wang H.; Cui Z.; Huang W. E.; Ye H. A Single-Cell Raman-Based Platform to Identify Developmental Stages of Human Pluripotent Stem Cell-Derived Neurons. P Natl. Acad. Sci. Usa 2020, 117 (31), 18412–18423. 10.1073/pnas.2001906117. PMC7414136 32694205

[ref34] Pavillon N.; Smith N. I. Non-Invasive Monitoring of T Cell Differentiation through Raman Spectroscopy. Sci. Reports 2023, 13 (1), 3129 10.1038/s41598-023-29259-8. PMC9947172 36813799

[ref35] Chaudhary N.; Nguyen T. N. Q.; Cullen D.; Meade A. D.; Wynne C. Discrimination of Immune Cell Activation Using Raman Micro-Spectroscopy in an in-Vitro & Ex-Vivo Model. *Spectrochimica Acta Part A Mol*. Biomol. Spectrosc. 2021, 248, 119118 10.1016/j.saa.2020.119118. 33214105

[ref36] Machidon A. L.; Pejović V. Deep Learning for Compressive Sensing: A Ubiquitous Systems Perspective. Artif. Intell. Rev. 2023, 56 (4), 3619–3658. 10.1007/s10462-022-10259-5.

[ref37] Wang L.; Zhang T.; Fu Y.; Huang H. HyperReconNet: Joint Coded Aperture Optimization and Image Reconstruction for Compressive Hyperspectral Imaging. IEEE Trans. Image Process. 2019, 28 (5), 2257–2270. 10.1109/TIP.2018.2884076. 30507509

[ref38] Kim C.; Park D.; Lee H.-N. Compressive Sensing Spectroscopy Using a Residual Convolutional Neural Network. Sensors 2020, 20 (3), 594 10.3390/s20030594. 31973148 PMC7037334

[ref39] Nordberg M.; Hollmann L.; Landström L.; Gustafsson D. Reconstruction of Compressed Sensing Raman Imaging Using Machine Learning. In Electro-Optical and Infrared Systems: Technology and Applications XVIII and Electro-Optical Remote Sensing XV, 2021; Vol 118660B, DOI: 10.1117/12.2600179.

[ref40] Calderbank R.; Jafarpour S.; Schapire R. Compressed Learning: Universal Sparse Dimensionality Reduction and Learning in the Measurement Domain. Technical Report 2009.

[ref41] Adler A.; Elad M.; Zibulevsky M. Compressed Learning: A Deep Neural Network Approach. arXiv 2016. doi:10.48550/arxiv.1610.09615.

[ref42] Xuan V. N.; Loffeld O. A Deep Learning Framework for Compressed Learning and Signal Reconstruction. In 2018 5th International Workshop on Compressed Sensing applied to Radar, Multimodal Sensing, and Imaging (CoSeRa2018), 2018.

[ref43] Abdar M.; Pourpanah F.; Hussain S.; Rezazadegan D.; Liu L.; Ghavamzadeh M.; Fieguth P.; Cao X.; Khosravi A.; Acharya U. R.; Makarenkov V.; Nahavandi S. A Review of Uncertainty Quantification in Deep Learning: Techniques Applications and Challenges. Inf. Fusion 2021, 76, 243–297. 10.1016/j.inffus.2021.05.008.

[ref44] Lotfollahi M.; Naghipourfar M.; Luecken M. D.; Khajavi M.; Büttner M.; Wagenstetter M.; Avsec Ž.; Gayoso A.; Yosef N.; Interlandi M.; Rybakov S.; Misharin A. V.; Theis F. J. Mapping Single-Cell Data to Reference Atlases by Transfer Learning. Nat. Biotechnol. 2022, 40 (1), 121–130. 10.1038/s41587-021-01001-7. 34462589 PMC8763644

[ref45] Eilers P. H.; Boelens H. F. Baseline Correction with Asymmetric Least Squares Smoothing. Leiden University Medical Centre Report 2005, 1.1, 5.

[ref46] He S.; Zhang W.; Liu L.; Huang Y.; He J.; Xie W.; Wu P.; Du C. Baseline Correction for Raman Spectra Using an Improved Asymmetric Least Squares Method. Anal Methods-uk 2014, 6 (12), 4402–4407. 10.1039/C4AY00068D.

[ref47] Matveeva I.; Bratchenko I.; Khristoforova Y.; Bratchenko L.; Moryatov A.; Kozlov S.; Kaganov O.; Zakharov V. Multivariate Curve Resolution Alternating Least Squares Analysis of In Vivo Skin Raman Spectra. Sensors 2022, 22 (24), 9588 10.3390/s22249588. 36559957 PMC9785721

[ref48] Fukunaga K. Introduction to Statistical Pattern Recognition. 1990.

