# Supplementary material for: Neural Network-Based Filter Design for Compressive Raman Classification of Cells
Source: J Chem Inf Model. 2024 Jul 3;64(14):5402–12. doi: 10.1021/acs.jcim.3c01856 (PMC11267571; doi:10.1021/acs.jcim.3c01856)
Supplement: Supplementary file 1 — ci3c01856_si_001.pdf [file ci3c01856_si_001.pdf]

SUPPORTING INFORMATION

Neural network-based filter design for compressive Raman classification of cells

Stefan Semrau

Leiden Institute of Physics, Leiden University, Leiden, 2333 CA, The Netherlands

Current affiliation: New York Stem Cell Foundation Research Institute, NY 10019, USA

E-mail: semrau@physics.leidenuniv.nl

SUPPORTING FIGURES

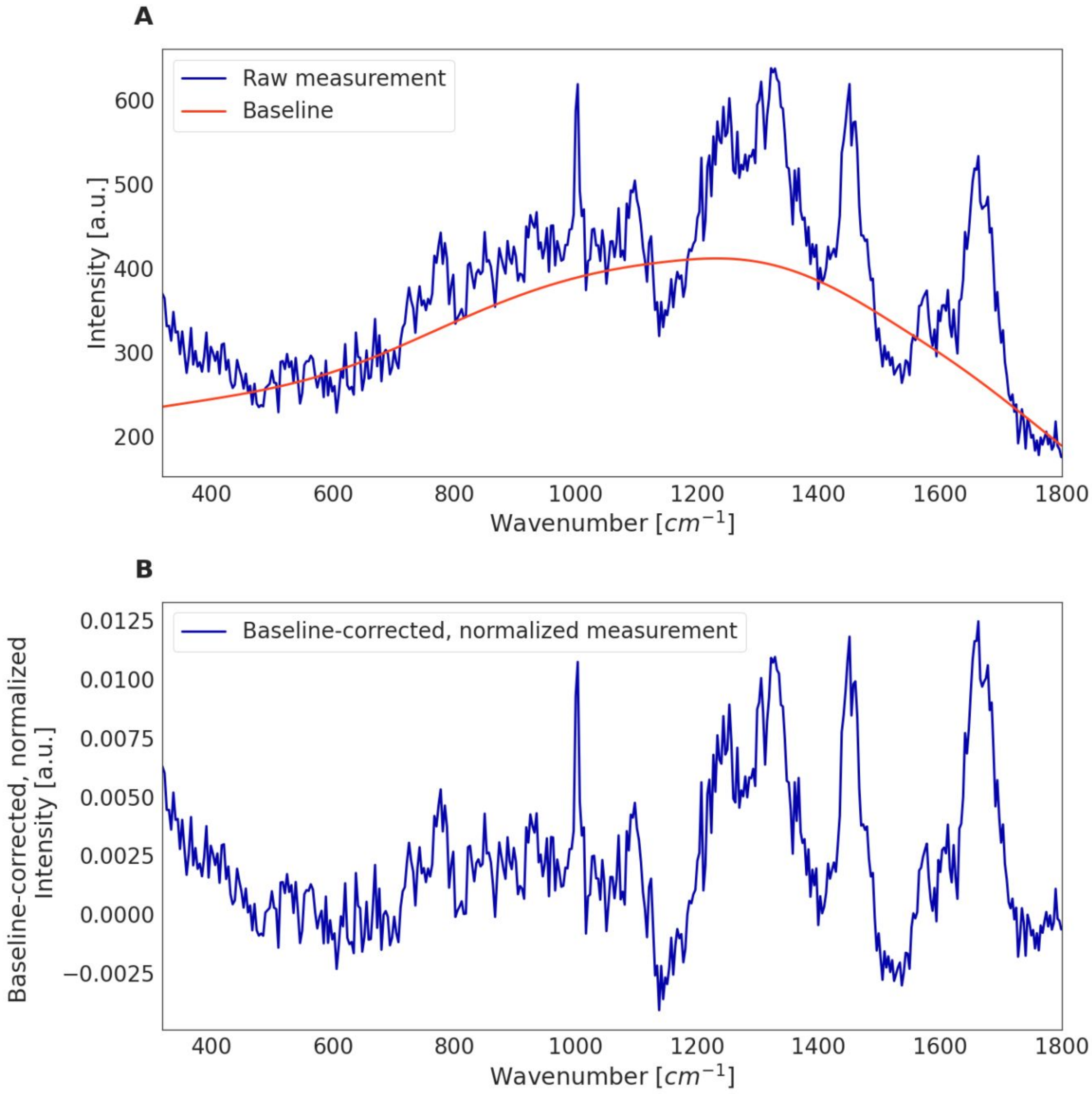

Figure S1 - Preprocessing removes a slowly varying baseline and normalizes spectra.
(A) A baseline calculated by asymmetric least-squares smoothing (red) is subtracted from the raw measurement (blue). (B) The baseline-corrected measurement shown is normalized to the sum of intensities over all considered wavenumbers.

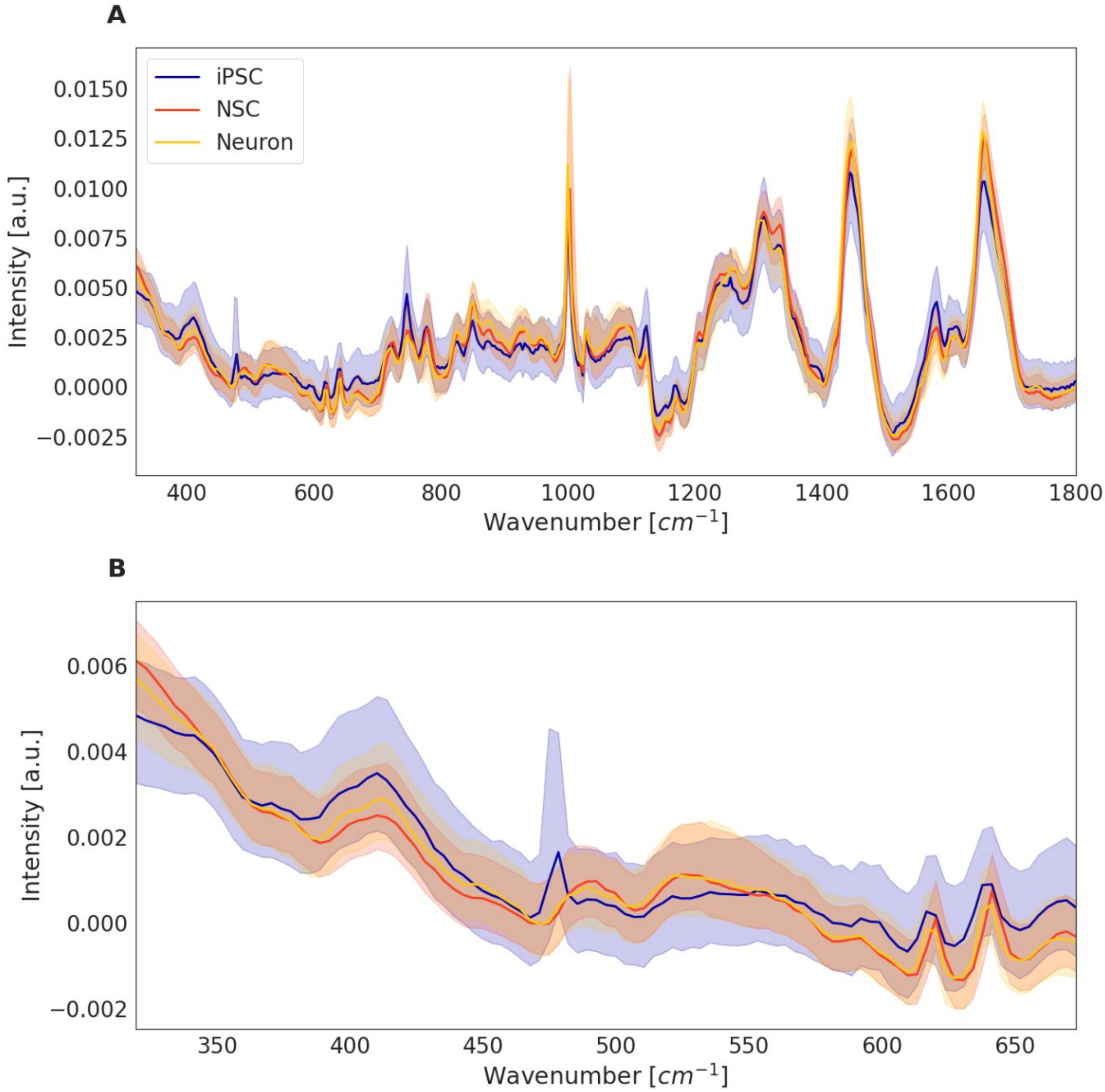

Figure S2 – Preprocessing reduces, but not eliminates, overlap of spectra.
Raman spectra after preprocessing (baseline removal and normalization). The solid lines show the medians per cell type. Error bands indicate mean absolute deviation calculated separately for positive and negative deviations. (A) Complete fingerprint region. (B) Zoom in on 319 cm^{-1} to 673 cm^{-1}.

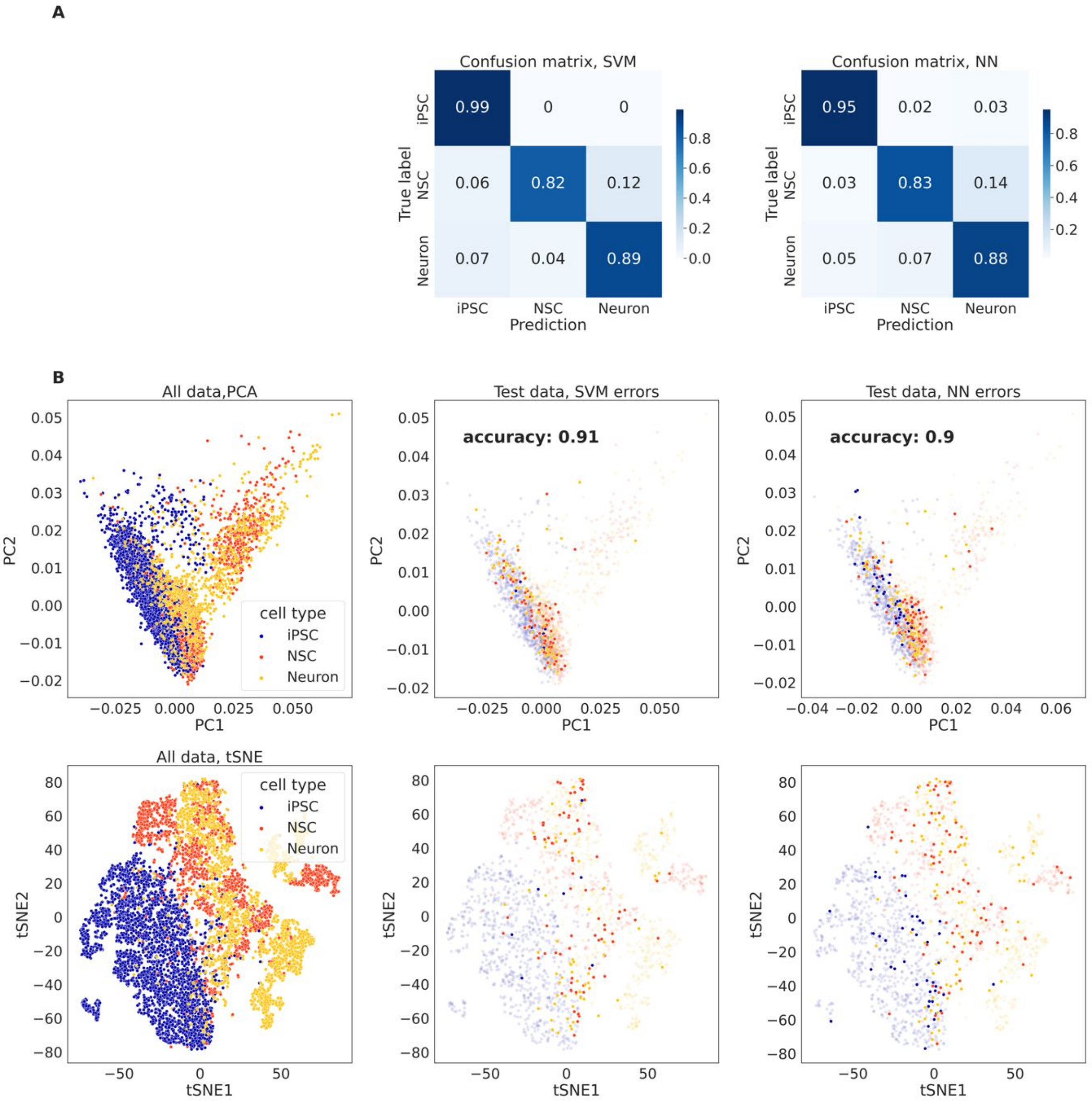

Figure S3 - Support vector machine and neural network classify cell types with high accuracy.

(A) Confusion tables of the SVM or NN classification of held-out test data. (B) Two-dimensional embeddings of the data. The first row shows the data projected on the first two principal components. The second row shows a two-dimensional t-distributed stochastic neighbor embedding (t-SNE). The second and third columns show only the test samples with the samples wrongly predicted by the SVM or NN highlighted.

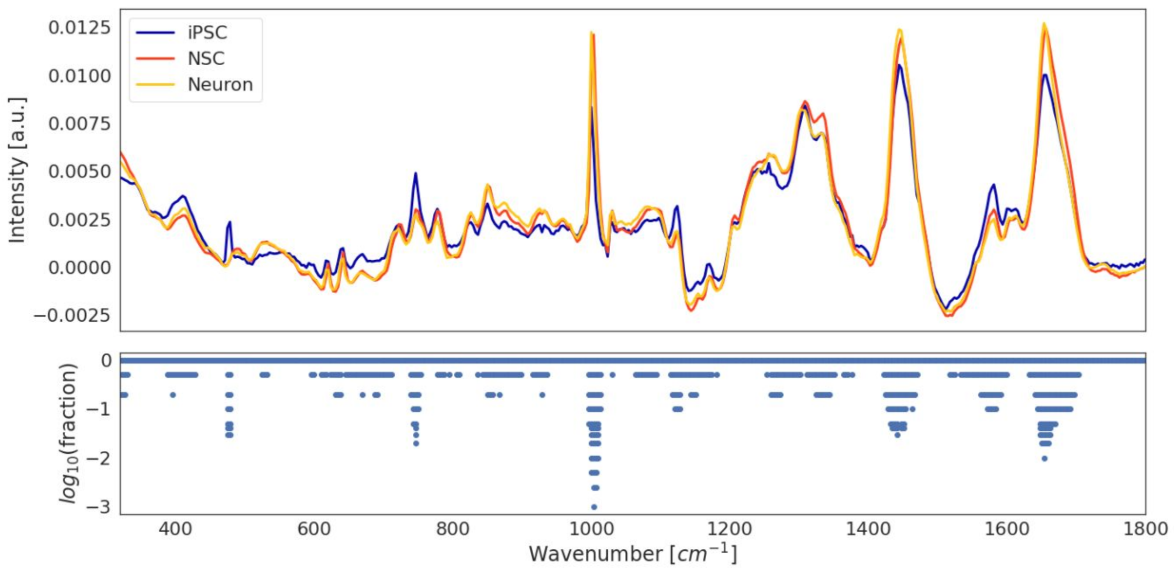

Figure S4 - Most informative Raman intensities are selected by variability across cell types. Top: Average Raman spectra for each cell type. Bottom: Wavenumbers with the most variable intensity across average spectra (standard deviation). The fraction of included wavenumbers is indicated on the y-axis.

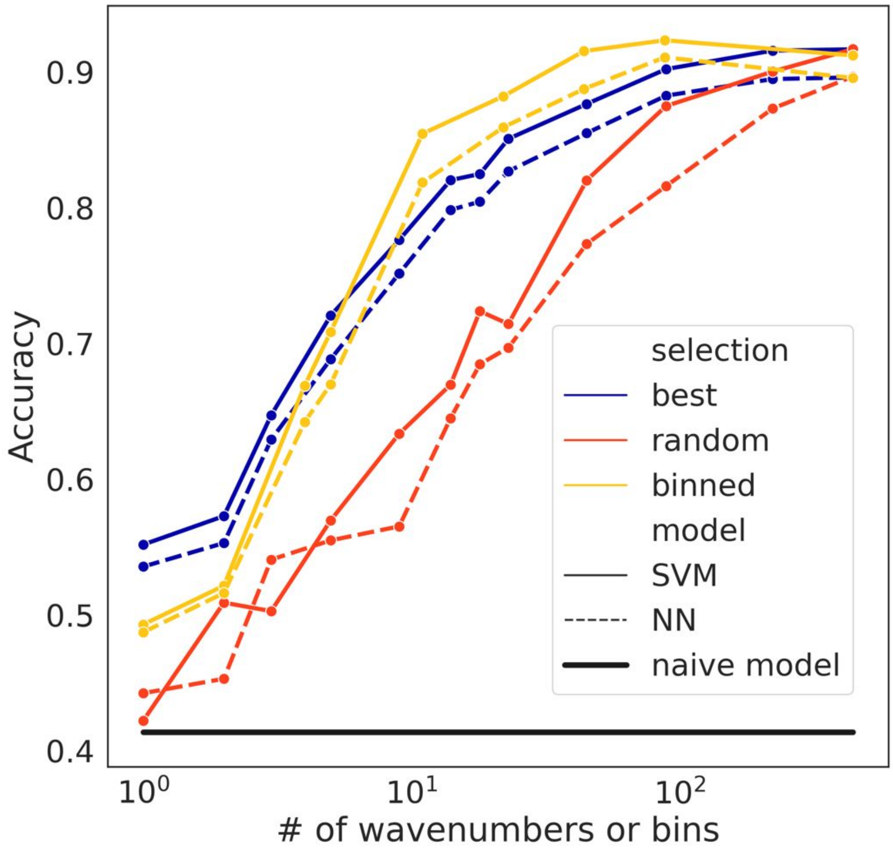

Figure S5 – For training on preprocessed spectra, classification accuracy increases with the number of included wavenumbers or wavenumber bins.
Accuracy on a held-out test set of for the support vector machine (SVM) or neural network (NN) model trained on preprocessed complete or subsetted spectra. For the results labeled 'best' or 'random', a subset of wavenumbers was chosen either based on the most variable wavenumbers ('best') or randomly ('random'). For the results labeled 'binned' Raman intensities were binned in equally sized wavenumber bins and averaged. The horizontal line indicates the accuracy of a naive model that always predicts the most frequent class.

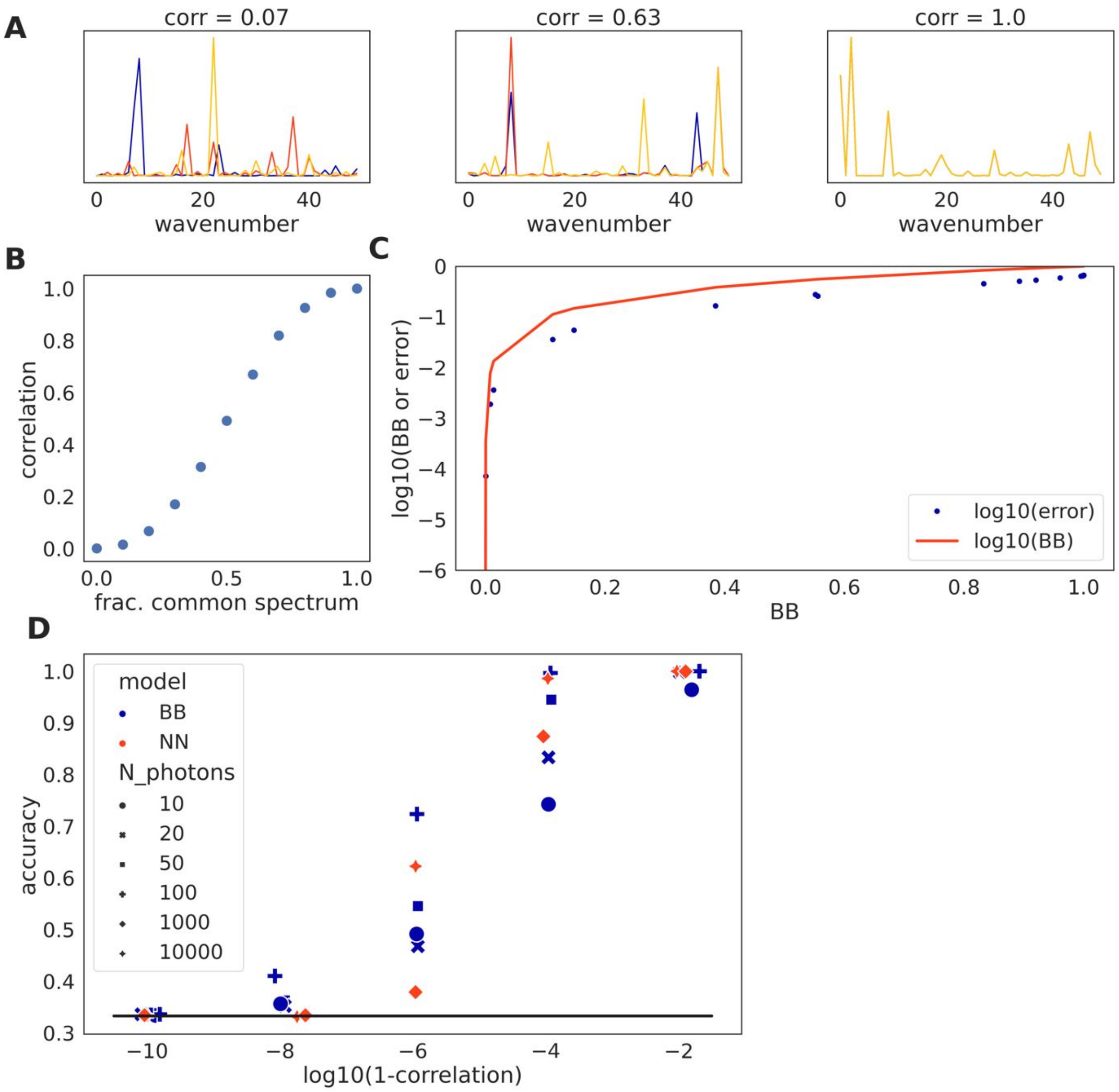

Figure S6 – Bhattacharyya bound-based optimization and neural network model perform similarly on simulated data.

(A) Examples of simulated spectra for 3 species, indicated by color, with different levels of Pearson correlation (corr). (B) Correlation between spectra versus the fraction of a shared, 'common' spectrum. Spectra of 3 species were created by simulating 4 spectra and defining one as a 'common' spectrum. To create correlation, a linear combination was calculated: (1-β)*spectrum + β*common spectrum, where β is the fraction reported on the x-axis. For β =1, all 3 spectra are identical, for β=0 the spectra are created from independent random processes. (C) Bhattacharyya bound (BB) and classification error of BB-based optimization for various levels of correlation and numbers of photons. The achieved error was always smaller than the BB bound. (D) Classification accuracy for the NN model or BB-based optimization for different correlations between the spectra and numbers of photons (N_{phot}). For the BB-based optimization, N_{phot} refers to the number of photons after the filter, for the NN model, it refers to the number of photons from the complete spectrum, before the filter. As the filters created by BB-based optimization have an approximate optical efficiency of 1% in our simulations, 1000 photons for the BB-based optimization are equivalent to 10000 photons for the NN model.

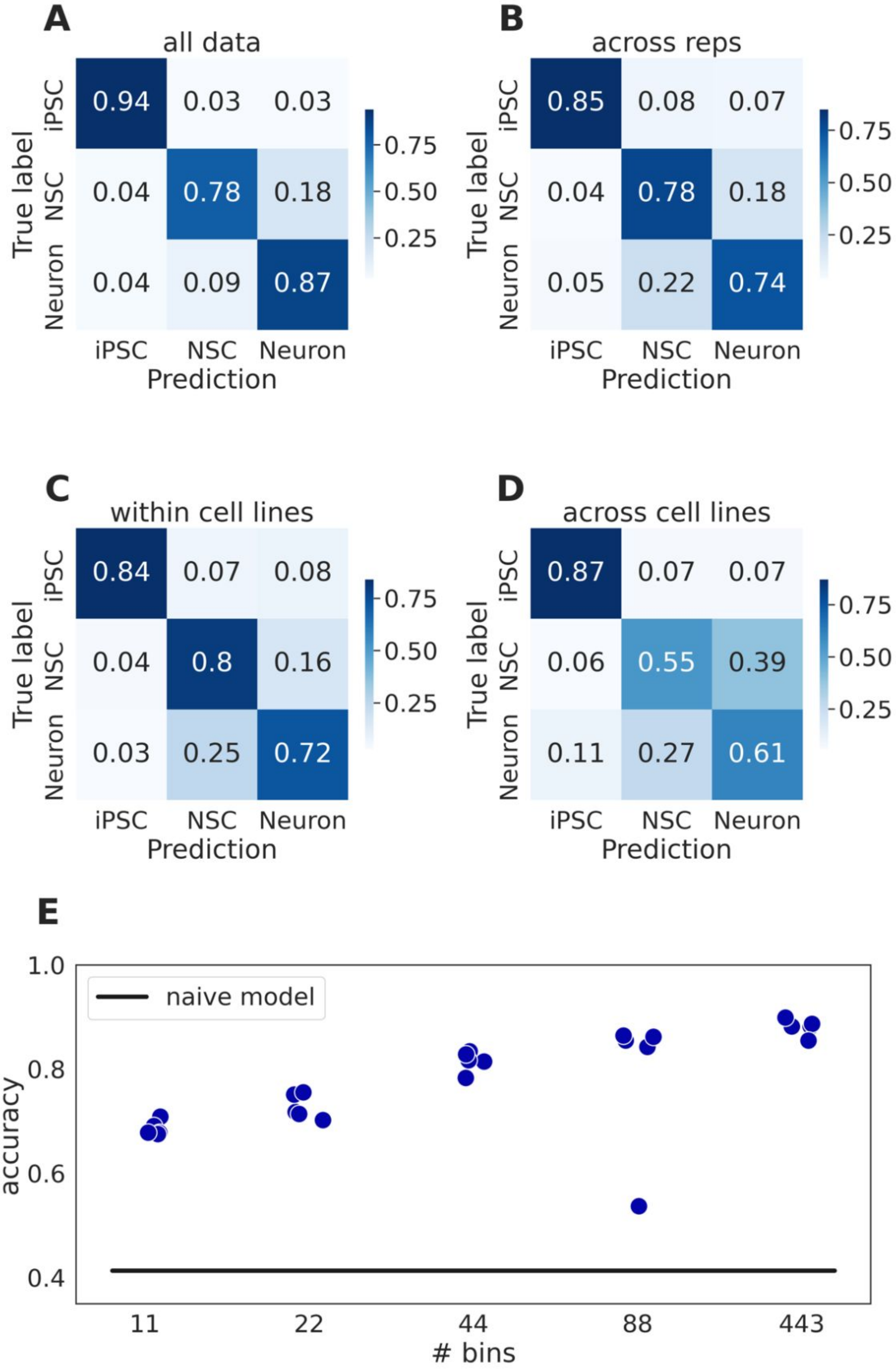

Figure S7 – Biological variability impacts prediction accuracy.
(A-C) Confusion tables for: training and testing using all data (A), training using two biological replicates and predicting the third, using all cell lines (B) or within individual cell lines (C) and training using two cell lines and predicting the third (D). (E) Classification accuracy of an NN model with 5 units in the first hidden layer (with layer normalization and binary weights) on held-out test data when trained on spectra averaged within bins (= consecutive intervals) of equal size.

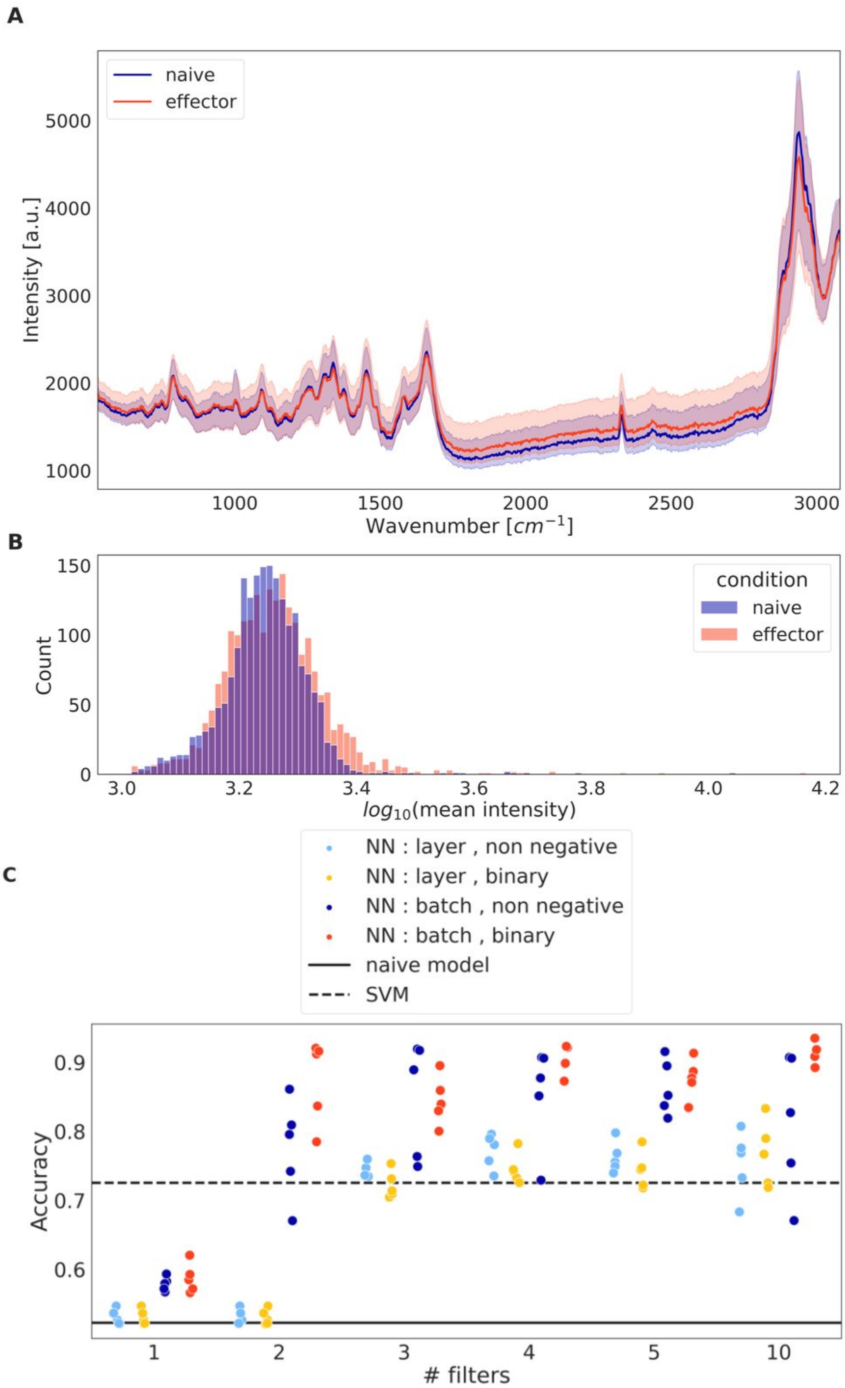

Figure S8 – Classification of T cell activation (Pavillon et al., Scientific Reports, 2023)

(A) Raw Raman intensities (no preprocessing). The data set contains spectra from CD4 T cells either left unstimulated (naïve, 2005 spectra) or activated for 24 h (effector, 2197 spectra). The solid lines show the median per condition for each wavenumber. The error bands indicate mean absolute deviations calculated separately for positive and negative deviations. (B) Mean intensities of individual raw Raman spectra. (C) Accuracy on held-out test sets for a neural network (NN) model with different numbers of units in the first hidden layer of the NN (= # filters). Two different constraints on the weights in the first hidden layer were used (either 'non-negative' or 'binary') as well as two types of normalization ('layer' or 'batch' normalization) after the first hidden layer. The 5 data points shown for each choice of parameters (# filters, constraint, normalization) correspond to 5 different splits of the data into training and test set for the NN model. The dashed horizontal line indicates the accuracy of a support vector machine trained on raw Raman spectra and the solid horizontal line corresponds to a naïve model that always predicts the most frequent class.

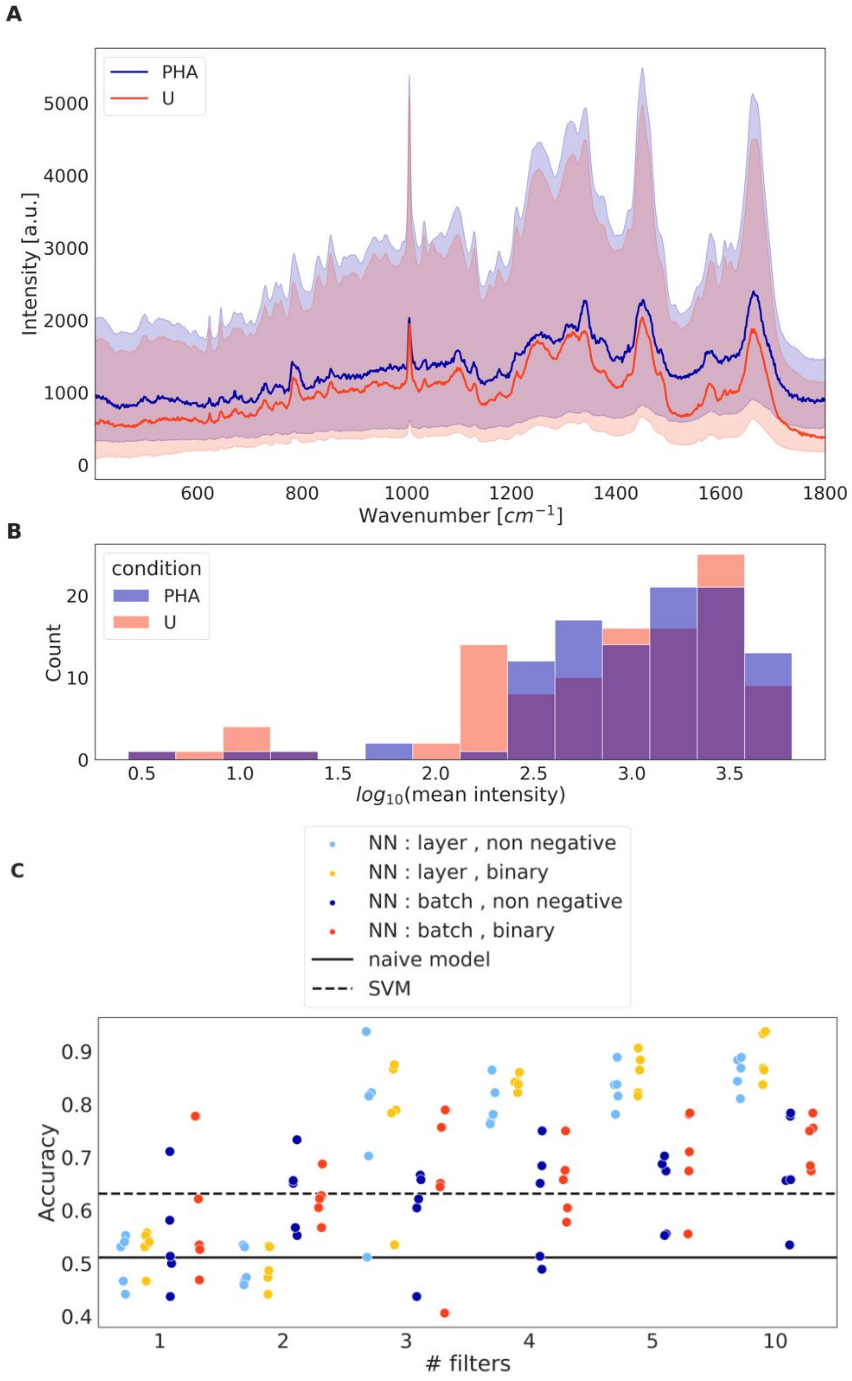

Figure S9 – Classification of T cell activation (Chaudhary et al., Spectrochimica Acta Part A: Molecular and Biomolecular Spectroscopy, 2023)

(A) Raw Raman intensities (no preprocessing). The data set contains spectra from T cells purified from blood either left untreated (U, 112 spectra) or treated for 72 h with phytohaemagluttinin (PHA, 107 spectra). The solid lines show the median per condition for each wavenumber. The error bands indicate mean absolute deviations calculated separately for positive and negative deviations. (B) Mean intensities of individual raw Raman spectra. (C) Accuracy on held-out test sets for a neural network (NN) model with different numbers of units in the first hidden layer of the NN (= # filters). Two different constraints on the weights in the first hidden layer were used (either 'non-negative' or 'binary') as well as two types of normalization ('layer' or 'batch' normalization) after the first hidden layer. The 5 data points shown for each choice of parameters (# filters, constraint, normalization) correspond to 5 different splits of the data into training and test set for the NN model. The dashed horizontal line indicates the accuracy of a support vector machine trained on raw Raman spectra and the solid horizontal line corresponds to a naïve model that always predicts the most frequent class.

A

B

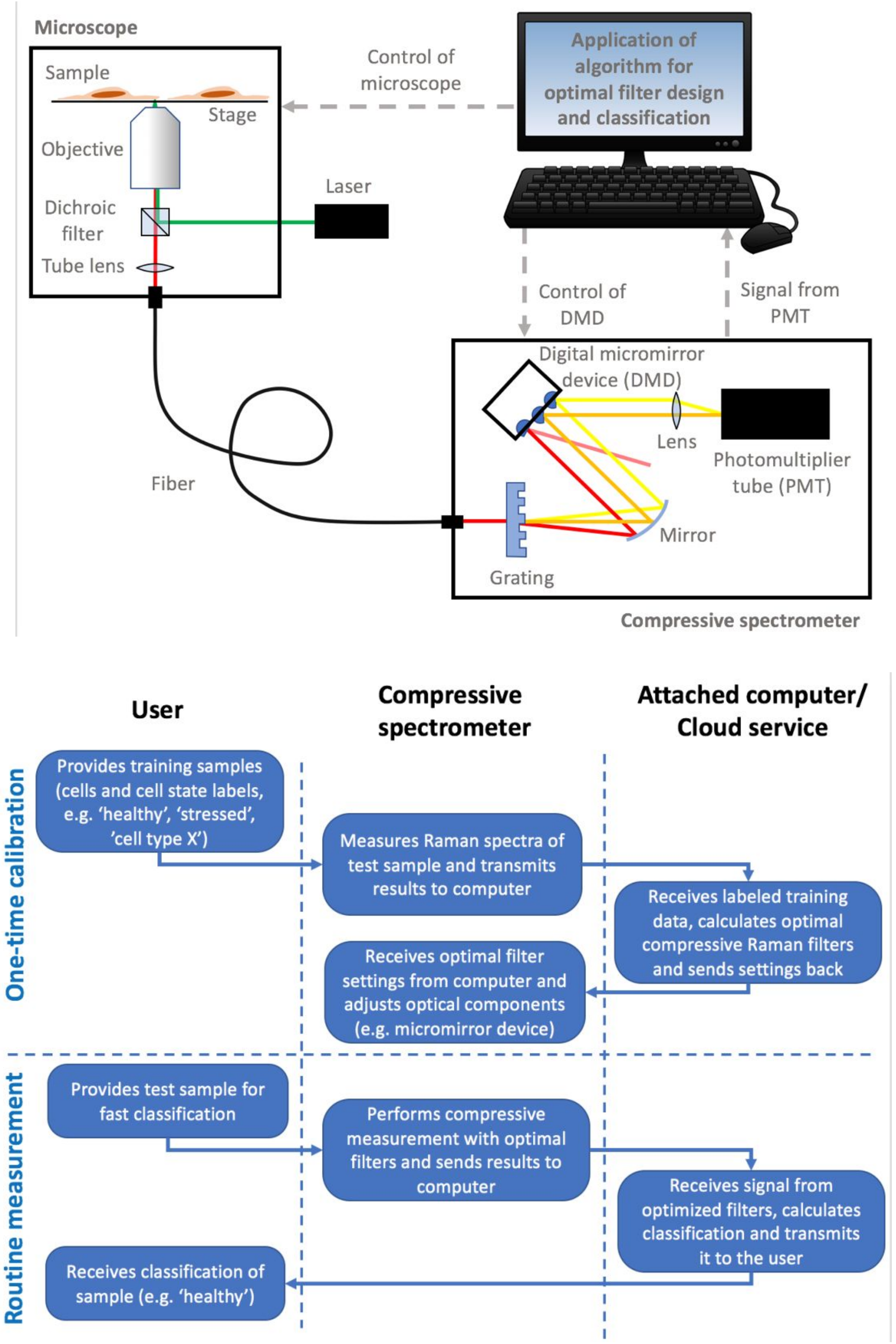

Figure S10 – Schematic of compressive Raman spectrometer and calibration workflow
(A) Schematic of a possible compressive Raman spectrometer setup (B) Workflow of spectrometer calibration and routine measurements

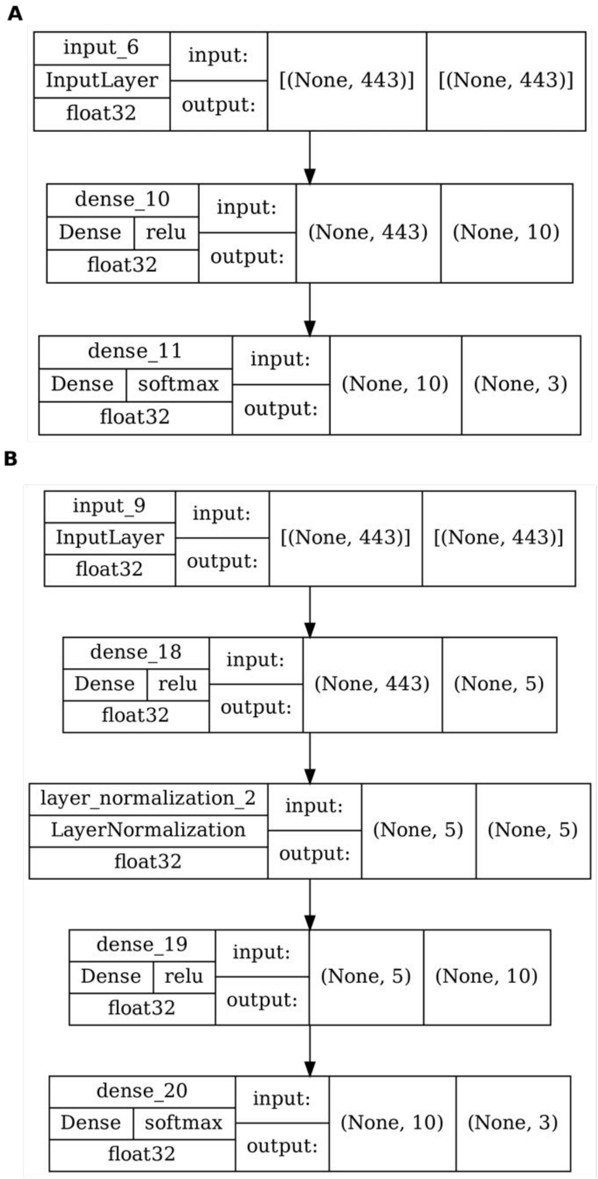

Figure S11 – Neural network models

Neural network models visualized by the *plot_model* function from *tensorflow*. (A) Model A, used for classification of preprocessed spectra. (B) Model B, used for classification of raw spectra and optimal filter design by introducing a binarity constraint on the weights of the first hidden layer. An example with 5 units in the first hidden layer is shown.

SUPPORTING TABLES

Cell type	Cell line	Rep	Date	Cells	Measure ments
iPSC	010S-1	1	181129	20	534
iPSC	010S-1	2	181130	20	495
iPSC	010S-1	3	181129	20	531
iPSC	014S-10	1	181129	20	424
iPSC	014S-10	2	181130	20	366
iPSC	014S-10	3	181130	20	327
iPSC	SB-AD3-1	1	181129	20	437
iPSC	SB-AD3-1	2	181129	20	389
iPSC	SB-AD3-1	3	181130	20	347
NSC	010S-1	1	190117	20	358
NSC	010S-1	2	190117	20	322
NSC	010S-1	3	190117	20	299
NSC	014S-10	1	190117	20	259
NSC	014S-10	2	190117	16	203
NSC	014S-10	3	190305	20	235
NSC	SB-AD3-1	1	190305	20	236
NSC	SB-AD3-1	2	190305	20	217
NSC	SB-AD3-1	3	190305	20	213
Neuron	010S-1	1	190305	20	316
Neuron	010S-1	2	190305	20	407
Neuron	010S-1	3	190305	20	405
Neuron	014S-10	1	190108	20	500
Neuron	014S-10	2	190108	20	394
Neuron	014S-10	3	190108	20	341
Neuron	SB-AD3-1	1	190108	20	250
Neuron	SB-AD3-1	2	190108	20	266
Neuron	SB-AD3-1	3	190108	20	237

Table S1 – Breakdown of data set created by Hsu et al.

state	measurements
naive	2005
effector	2197

Table S2 – Breakdown of data set created by Pavillon et al.

treatment	measurements
U	112
PHA	107

Table S3 – Breakdown of data set created by Chaudhary et al.
